# Feeling safe in the context of digitalization in healthcare: a scoping review

**DOI:** 10.1186/s13643-024-02465-9

**Published:** 2024-02-08

**Authors:** Peter Minartz, Christine Maria Aumann, Carmen Vondeberg, Silke Kuske

**Affiliations:** Fliedner Fachhochschule Düsseldorf, University of Applied Science, Alte Landstr. 179, 40489 Düsseldorf, Germany

**Keywords:** Emotional safety, Psychological safety, Digitalization, Healthcare, Patient Safety, eHealth, Telemedicine

## Abstract

**Background:**

Digitalization in healthcare and society can be challenging, particularly for people who have limited digital experiences. New digital technologies can influence individuals’ perceived safety and well-being. In this study, we aimed to identify and analyze the literature on needs and influencing factors in the context of emotional and psychological safety and digitalization in healthcare.

**Methods:**

A scoping review was conducted based on the PRISMA-ScR standard. The literature was searched based on the databases Medline via PubMed, PsycINFO via Ovid, and CINAHL via EBSCO. Literature was included after a review of the titles, abstracts, and full texts published in English or German in the last 5 years (October 2017–September 2022). Eligible literature included definitions and descriptions of emotional and/or psychological safety and was related to digitalization in healthcare and was analyzed qualitatively via inductive content analysis. The findings were analyzed from ethical, psychosocial, legal, economic, and political perspectives.

**Results:**

A total of 32 publications were finally included thereof qualitative (*n* = 20), quantitative (*n* = 3), and mixed methods (*n* = 2) studies. Other included publications were systematic integrative reviews, scoping reviews, narrative reviews, white papers, and ethical statements. Of these publications, four qualitative studies focused on emotional or psychological safety in the context of digital technology use in healthcare as a primary research aim. Most literature has shown that perceived safety is influenced by perceived changes in healthcare, digital (health) literacy, the design of digital technology, and need orientation. The needs identified in this context overlap strongly with the influencing factors. A low or high perceived safety has an impact on users’ thoughts and actions.

**Conclusion:**

The importance of emotional safety in the context of digital technologies in healthcare is growing, while psychological safety seems to be underrepresented. The interaction between the influencing factors and the need to feel safe leads to considerations that can affect user behavior and have far-reaching outcomes for the implementation of digital technology in healthcare.

**Systematic review registration:**

Open Science Framework Registries on 16 December 2022 https://doi.org/10.17605/OSF.IO/HVYPT.

**Supplementary Information:**

The online version contains supplementary material available at 10.1186/s13643-024-02465-9.

## Background

In recent years, digital transformation has become increasingly important in the healthcare sector [1]. From a societal perspective, digital transformation offers both societal and healthcare benefits and is considered a social task given the various challenges related to demographic change [2]. In this context, digitalization is necessary to ensure future high-quality medical care [3]. Digitalization is defined as an improvement process involving the use and application of digital technologies (DTs) such as robotics, home monitoring, or telehealth in individual, organizational, or societal contexts [1, 3]. Generally, research in the context of safety and digitalization is focused on data security [4]. The comprehensive context of perceived safety and digitalization is minor considered [5]. DTs are intended to support people and ensure independence, participation, and safety [2, 6, 7], and safety, trust, and acceptance are core requirements in the increasing demand for the use of DTs in healthcare [8]. New DTs can influence perceptions of well-being and safety [9, 10]. From an ethical and psychosocial perspective, DTs must protect people from physical and psychological harm and provide a feeling of safety [11, 12]. A feeling of safety refers to freedom from the threat of physical or emotional harm and is a basic human need [13]. Feeling safe refers to the perception of how an individual is affected by external threats but also having an inner sense of safety, which is one’s ability to authentically express one’s inner strength and overall well-being [14]. From a patient safety perspective, this dimension is described as emotional safety [15] and is located on a continuum of feeling safe and not feeling safe in relation to inner and outer conditions [16]. In the context of DTs, feeling safe refers to the perceived level of danger compared to the perceived level of comfort when interacting with DTs [17].

Emotional safety can have a decisive influence on attitudes toward change and can thus be a key decision criterion for or against innovation implementation [18]. The concept of perceived safety is also related to psychological safety in the work environment [11, 19]. It can be understood as a sense of feeling safe in professional settings, e.g., healthcare teams, where it is allowed and sometimes even desired to make mistakes, to try new things [20], and consequently to feel safe in a rapidly changing work environment in terms of developing, learning and effective performance [21].

Traditionally, emotional safety is attributed to healthcare recipients (e.g., patients) [15], and psychological safety to healthcare providers [22]. In this context, perceived safety plays a role in the implementation of DTs [6]. Digitalization aims to make healthcare more efficient, although there is a decline in efficiency due to limited acceptability and trust [8]. In this context, it is emphasized that if the relevance of the innovation is recognized by the users, an increased acceptability is observed [23]. Perceived safety can also contribute to the acceptability of different DTs [11]. Accordingly, acceptability can be an important factor in the successful implementation of an intervention for both healthcare recipients and providers [24]. Higher acceptability increases patient adherence and leads to improved clinical outcomes for healthcare providers, which in turn influences the scope and use of new innovations [23]. The introduction of a new DT can lead to skepticism or uncertainty about its use [25], which in turn can lead to lower perceptions of safety [11]. Despite the importance of feeling safe in relation to digitalization and DT use in healthcare, there is no systematic overview of the literature that considers needs and influencing factors (IFs) in the context of feeling safe, digitalization, and DT use in healthcare. Specifically, an overview that considers ethical, psychological, legal, economic, and political aspects is lacking. Therefore, the aim of this scoping review was to identify and analyze the literature considering needs and IFs in the context of emotional and psychological safety, digitalization, and DT use in healthcare.

## Methods

This scoping review was conducted according to the ‘PRISMA Extension for Scoping Reviews (PRISMA-ScR) [26]. Additionally, grey literature was searched based on relevant criteria [27]. Data extraction was carried out according to the Cochrane guidelines [28] and the Joanna Briggs Institute (JBI) guidelines [29]. The review protocol is registered on the Open Science Framework, 16 December 2022 https://doi.org/10.17605/OSF.IO/HVYPT.

### Data sources

The published literature was identified by searching the following databases: MEDLINE via PubMed, PsycINFO via OVID, and CINAHL via EBSCO. In addition, grey literature was searched using Google, Google Scholar, the union catalog of university libraries, the electronic journal library, and the websites of agencies that fund, report, or conduct literature for healthcare research in Germany (e.g., the Federal Ministry of Education and Research or the Federal Ministry of Health). In addition, we used backward citation tracking for all included publications and forward citation tracking for studies with the primary outcome of emotional safety.

### Search strategy

The search strategy comprised both controlled vocabulary, such as the National Library of Medicine’s Subject Headings (MeSH), and keywords. The search strategy followed the outlined criteria for systematic qualitative research (PICo = population, the phenomenon of interest, and the context) [30]. One reviewer developed the search strategy, which was peer-reviewed using the Peer Review of Electronic Search Strategies checklist (PRESS) [31] (see Additional file 1).

### Inclusion and exclusion criteria

Literature published in English or German between 01 October 2017 and 30 September 2022 was searched. The search period was chosen because our pre-search showed that the research topic was increasingly published since 2017, in the line of evolved technological advances. For inclusion, publications had to address emotional and/or psychological safety in relation to digitalization and/or DT use in healthcare. All types of studies, reviews, and expert reports, e.g., expert-based approaches, were included. A precise type of DT was not relevant for inclusion or exclusion. A clear description or definition of emotional or psychological safety had to be provided in the literature; otherwise, the literature was excluded.

### Study selection

The study selection based on the inclusion and exclusion criteria was pretested by two reviewers by title-abstract screening of the first 100 records. Different voting was discussed for clarification. Duplicates were searched and removed by Citavi automatically and manually. Two reviewers independently double-checked the titles and abstracts of all systematically identified studies, which were rated as ‘include’, ‘unclear’, or ‘exclude’. For records categorized as ‘include’ or ‘unclear’, the full text was also independently screened by two reviewers for final eligibility. In line with the PRISMA-ScR guidelines [26], we did not apply a methodological quality appraisal; however, we described the study designs and methods. Additionally, grey literature was selected by two reviewers based on the title and abstract, if available, and subsequently by full text.

### Data extraction

Data were extracted by one reviewer and independently checked for accuracy by a second reviewer. Disagreements were resolved by discussion until a consensus was reached. Data extraction was performed according to the research question and by described methods [28, 29]. The following data were extracted: first author, year of publication, design (methods), participants (sample), setting (country of origin), DTs in healthcare, and objective.

### Data analysis and synthesis

The data were analyzed in accordance with the methods of Whittemore and Knafl [32], including data reduction, data display, data comparison, and conclusion drawing and verification. Data extracted from grey and scientific literature were integrated into the analysis process, and a qualitative content analysis [33] was conducted. Eligible literature was first read several times to gain an initial understanding of the content of the entire material. The primary sources were divided into subgroups of emotional and psychological publications. Units of meaning (words, sentences, or paragraphs) were identified for the purpose of the study. The meaning units were condensed and coded and initially sorted into facilitating and inhibiting factors or those that were addressed both. The resulting (sub) categories were then tabulated and compared considering the DT and the target group (healthcare recipients, healthcare providers, or informal caregivers). The data were compared to identify patterns, themes, or relationships. The resulting categories were synthesized into core dimensions of influencing factors (CDIFs) based on commonalities and differences. The CDIFs were located based on the results in a matrix that allows a multidimensional view at the micro, meso, and macro levels. In line with this approach, the needs and outcomes of feelings of safety were analyzed. Finally, the included literature was reviewed for ethical, psychosocial, legal, economic, and political perspectives. Coding was carried out by one reviewer and checked by a second reviewer. Finally, all results were critically discussed by the research team, and conclusions were drawn.

## Results

**Fig. 1:**
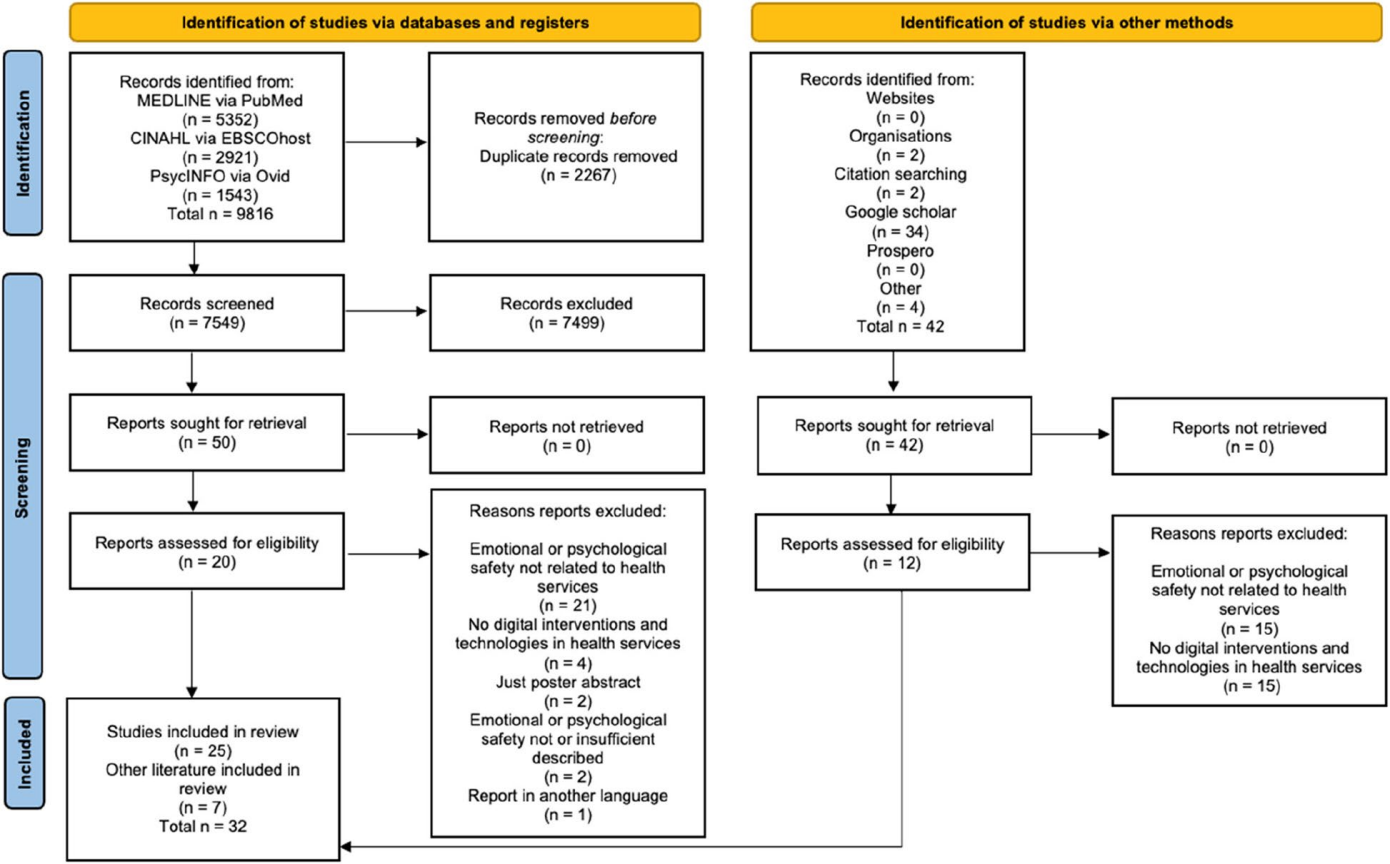
PRISMA 2020 flow diagram inspired by Page et al. [34]

A total of 9816 hits were identified from the scientific databases in addition to 42 hits from grey literature (see Fig. 1). Finally, 32 publications, including grey literature, were considered eligible (see Table 1). Of these 25 were studies.

**Table 1:** Characteristics of the identified publications

Author (years)	Design (methods)	Sample	Setting	Digital technology	Objective
**Qualitative studies**					
Åkerlind et al. (2018)^a^ [6]	Exploratory (semi structured individual interviews)	*N* = 12 participants (older patients who had been provided with eHomecare and 8 relatives)	At home (Sweden)	Night camera (for supervision at night); (portable) videophone (for daily check-ups, social interactions, and reminders); electronic mailbox (for reminders and information by SMS)	To extend descriptions of how older patients with granted eHomecare and their relatives understand safety, and further to describe how they experience safety in everyday life
Bajaj et al. (2017) [35]	Design n.f.d. (observation)	*N* = 14 NYC Health + Hospital facilities (including 12 hospital-based prenatal clinics/2 outpatient clinics)	In Hospitals (USA)	Incognito patients embedded within electronic health record (Zika Simulation Drills)	To highlight challenges and solutions to implementation of an incognito embedded participant simulation program across a large healthcare system to enhance preparedness for a public health threat (Zika Virus)
Bhattacharya et al. (2021) [36]	Exploratory (interviews and surveys)	*N* = 18 participants (*N* = 10 mental health clinicians; *N* = 8 teens with depression)	Internet (USA)	Internet-based groups on Slack	To understand needs and challenges that patients face in depression management and involve them in the design process of a remote intervention that uses asynchronous remote communities, to understand the benefits and challenges of adapting behavioural activation to an internet-based platform that supports the asynchronous remote community approach as a delivery tool for teen depression management
Demiris et al. (2019) [37]	Exploratory (analysis of coded transcripts of in-depth interviews)	*N* = 171 participants (*N* = 88 adults 60 years and older; *N* = 56 identified by older adults as friends and family (involved in health/health information management) *N* = 27 clinicians (physicians, nurses, social workers)	At home (USA)	Personal health information management tools	To explore how personal health information management can be a facilitator for patient safety in the home
Dixon et al. (2022) [38]	Exploratory (semi structured telephone interviews), fictional vignette-based scenarios)	*N* = 18 participants (General Practitioners)	At home (England)	Remote consultation during the COVID-19 pandemic	To explore GP perspectives and concerns about safeguarding practice during the pandemic, focusing on challenges and opportunities created by remote consultation
D’Onofrio et al. (2019) [39]	Descriptive (prestructured questionnaire)	*N* = 53 participants (*N* = 36 caregivers (21 professionals; 15 informal; *N* = 17 adults (> 60 years))	Setting n.f.d (Italy and Japan)	Robotic solutions for ageing	To provide a pilot qualitative analysis of the needs of elderly people and their caregivers when exposed to conversational activities with robots with prioritisation from end-user perspectives
Donovan et al. (2021) [40]	Explorative (semi structured interviews)	*N* = 35 participants (patients)	At home (USA)	Telehealth	To assess patients’ experiences receiving sleep care by telehealth
Hylén et al. (2022) [41]	Descriptive (semi structured telephone interviews)	*N* = 25 participants (parents)	At home (Sweden)	Specific mobile eHealth tablet (for digital communication between parents and healthcare providers during the family’s transition from hospital to home)	To analyze access to healthcare as perceived by parents when caring for their child at home, with conventional care supported by eHealth following paediatric surgery or preterm birth
James et al. (2021) [42]	Descriptive (semi structured telephone interviews)	*N* = 25 participants (primary healthcare nurses)	At home (Australia)	Telehealth during the COVID-19 pandemic	To explore the experiences of Australian primary healthcare nurses in the use of telehealth during COVID-19
Johannessen (2021)^a^ [43]	Exploratory (focus groups, individual/semi structured interviews)	*N* = 29 participants (*N* = 10 homecare-professionals; *N* = 10 managers; *N* = 9 older telecare user)	At home (Norway)	Telecare	To contribute to more insight and knowledge regarding patient safety and perception of safety when telecare is used, by exploring the perceptions and experiences
Karlsen et al. (2019) [44]	Hermeneutic (interviews and follow-up interviews)	*N* = 25 participants, older adults (*N* = 18, follow up *N* = 15); *N* = 7 close family caregivers)	At home (Norway)	Telecare/telemonitoring	To obtain a deeper understanding of the persistent use of telecare
Lounsbury et al. (2021) [45]	Descriptive (participants listening to recorded stories, writing their thoughts on a card)	*N* = 352 participants (no special type)	Setting n.f.d (UK)	Health care data sharing	To explore the views of the public, particularly their hopes and concerns, around healthcare data sharing
Lynch et al. (2022)^a^ [46]	Post-phenomenological (semi structured interviews/ethnographical case studies with observations)	*N* = 47 participants (patients)	At home (UK)	Home monitoring and communication kit (internet-enabled tablet computer and Bluetooth-enabled blood pressure and heart rate monitor, and weighing scales)	To explore users’ different ways in which reassurance is experienced with different types of technology-enabled care
Maier et al. (2021) [47]	Phenomenological (open-ended online questions, follow-up interviews)	*N* = 25 participants (patients) *N* = 2 participants (patients) in follow-up interviews	At home (USA)	Teletherapy during the COVID-19 pandemic	To explore the lived experiences of individuals in teletherapy, specifically those engaging in teletherapy with a romantic partner or family member
Malmgren Fänge et al. (2019) [48]	Exploratory (semi-structured interviews)	*N* = 30 participants (9 people with dementia; 21 family members)	At home (Sweden)	Sensor-based technology (home-leaving-, smoke-, water leak-, door-, window-, motion-based bed- and automatic light sensors)	To evaluate the effects of sensor-based technology on independence among people with dementia and care- giver stress among their family members
Nakrem et al. (2018) [2]	Exploratory multicase (semi structured interviews)	*N* = 21 participants (health care professionals)	At home (Norway)	Digital medicine dispensers	To explore how home healthcare professionals had experienced the introduction of digital medicine dispensers and their influence on patient-caregiver relationships
Nyholm et al. (2021)^a^ [3]	Descriptive (video vignette and semi-structured interviews)	*N* = 12 participants (no special type)	Hospital (Finland)	Robotics	To illuminate users’ sense of security with humanoid robots in healthcare
Raja et al. (2022) [9]	Reflective Lifeworld Research design (in-depth interviews)	*N* = 13 participants (75 or older)	Setting n.f.d (Norway)	Digitally led healthcare	To clarify the phenomenon of sense of dignity experienced in older adults, concerning how their expectations and needs are met within the context of digitally led healthcare in Norway
Strand et al. (2022) [12]	Descriptive participatory (Employing a framework of complex interventions and interviews)	*N* = 11 participants (*N* = 8 parents; *N* = 3 nurses)	At home (neonatal care) (Sweden)	Technical development of an eHealth application for computer tablets (video and information exchange)	To develop an eHealth device supporting the transition from hospital to home for parents with a preterm-born child in Sweden using participatory design
Winberg et al. (2021) [49]	Descriptive (focus group interviews)	*N* = 16 participants (people with a neurological disability)	At home (Sweden)	Apps to facilitate self-management in everyday life	To describe how people living with a neurological disability, e.g., multiple sclerosis, Parkinson’s disease, stroke, using apps for facilitating self-management
**Quantitative studies**					
Doorley et al. (2020) [50]	Descriptive (ecological momentary assessment and day reconstruction method)	*N* = 428 participants (people with varying levels of social anxiety; 3172 observations	Setting n.f.d (USA)	Digital communication	To better understand the influence of social anxiety on communication behaviours and how people with elevated social anxiety feel when they are communicating digitally versus face-to-face
Hughes et al. (2021) [51]	Descriptive case‒control-study (prepost surveys, observations)	*N* = 50 participants (surgical teams)	MASCAL simulation drill (USA)	Telemedical device during a mass casualty event simulated training	To examine the positive and/or negative impacts of telemedicine on teamwork in teams responding to mass casualty events
Lang et al. (2022) [52]	Longitudinal bicentric interventional (multiple quantitative methods of data collection/analysis)	*N* = 177 participants (multimorbid older patients) + e.g., general practitioners, therapists, social services	At home (Germany)	Telemonitoring	To develop an information/communication platform for an intersectoral networking. The development and testing of this telemonitoring applications aimed to optimise patients’ healthcare
**Mixed methods studies**					
Ninatti and Piergiovanni (2019) [53]	Mixed methods: exploratory qualitative (In-person interviews) /descriptive quantitative (survey)	*N* = 9 participants (different professionals) *N* = 82 participants (different professionals)	Setting n.f.d (Italy)	Digital technologies in general for the treatment of eating disorders	To explore the opportunities provided by digital technologies for eating disorder treatment and to explore factors of the intention to use digital technologies of the professional figures who treat eating disorders according to their impact on psychological well-being
Rogerson et al. (2019) [54]	Mixed methods: descriptive: quantitative (usage analysis) /qualitative (semi-structured interviews)	*N* = 19 stroke survivors who lived alone	At home (UK)	Monitors users’ activity in the home and uses machine learning algorithms to detect activity changes; family member contacted if activity changes	To assess the feasibility and acceptability of a special smart home system for stroke survivors
**Reviews**					
Kupfer and Mayer (2019) [55]	Narrative review	Children and adolescents	At home (Germany)	Online (blended) counselling	To identify research on online counselling, established primarily in English-speaking countries (Australia, UK), referenced to confidentiality and emotional security. The establishment of a short-term counselling relationship, autonomy, and a sense of control, and low-threshold access and exclusion as relevant themes
Salvador Vergès et al. (2022) [56]	Scoping Review	(n. a.)	Palliative care (Spain)	Telemedicine	To describe the current use of telemedicine in palliative care and assess stakeholders’ views on the initiatives that have been implemented worldwide regarding digital service standards
Van Olmen (2022) [57]	Narrative Review	(n. a.)	Setting n.f.d (Belgium)	E-Health interventions	To examine the mechanisms among e-Health interventions, self-management, and wellbeing
Widberg et al. (2020) [7]	Systematic integrative review	*N* = 397 participants (patients)	Palliative care (Sweden)	E-Health	To describe patients’ experiences of eHealth in palliative care
**Grey literature**					
German government's Data Ethics Commission (2019) [58]	Report (n. a.)	(n. a.)	Setting n.f.d (Germany)	Three topics: algorithm-based forecasting and decision-making, AI, and data	To provide ethical standards and guidelines for the protection of the individual, the preservation of social coexistence and the safeguarding and promotion of prosperity in the information age
German ethics council (2020) [59]	Statement “Robotics for good care” (n. a.)	(n. a.)	Setting n.f.d (Germany)	Robotics in healthcare	To provide a statement on the discussion of robotics in nursing care
Perry et al. (2021) [60]	White Paper	(n. a.)	At home (USA)	Telemedicine	To provide a framework for ensuring safe, equitable, person-centred telemedicine

^a^Feeling of safety as a primary aim of the study, *n.f.d.* not further defined, *n. a.* not applicable

Four qualitative studies [3, 6, 43, 46] of the final 32 eligible publications addressed perceived safety as a primary outcome, focusing on how (older) people perceived safety in everyday life when exposed to different DTs, e.g., camera surveillance and digital information exchange [6], telemonitoring and communication [46], telecare [43], and robotics [3], in their homes or hospitals. The remaining 28 publications (*n* = 21 studies) addressed the phenomenon as a secondary outcome (see Table 1).

Twenty-one of the 32 publications were published between 2020 and 2022 [3, 7, 9, 12, 15, 36, 38, 40–43, 45–47, 49–51, 56, 57, 59, 60], and 11 were published between 2017 and 2019 [2, 6, 35, 37, 39, 44, 48, 53–55, 58]. The identified studies used qualitative (*n* = 20) [2, 3, 6, 9, 12, 35–49], quantitative (*n* = 3) [50–52], or mixed-methods designs (*n* = 2) [53, 54]. The other publications included one systematic integrative review [7], one scoping review [56], two narrative reviews [55, 57], one white paper [60], and two ethical statements [58, 59]. In the majority, a home setting (*n* = 18) [2, 6, 12, 37, 38, 40–44, 46–49, 52, 54, 55, 60] was addressed. Eight publications [9, 39, 45, 50, 53, 57–59] did not assign the topic to a specific setting. The participants’ age groups ranged from children and adolescents to individuals of a very advanced age, including those with specific conditions (e.g., people living with dementia, neurological conditions, or social anxiety). Some studies distinguished between healthcare recipients (*n* = 16) [3, 6, 7, 9, 40, 41, 44, 46–50, 52, 54, 55, 57] and providers (*n* = 7) [2, 35, 38, 42, 51, 53, 56], while others included both perspectives (*n* = 5) [12, 36, 37, 39, 43] or examined the healthcare system in general (*n* = 4) [45, 58–60].

For all target groups, we found that a strong or low perceived safety could influence thoughts and actions (see Additional files 2 and 3); for example, strong perceived safety could lead to increased motivation to use DTs and enable individuals to stay at home alone and not move into a nursing home [6, 44], whereas low perceived safety could lead to fear of failure [9] and reduced capacity to participate effectively in one’s own disease management [37]. Regarding the implementation of DTs, we found that strong perceived safety increased the acceptance of DTs as an important factor when introducing new DTs [43, 48, 51], whereas low perceived safety resulted in the rejection of DTs [44, 60]. In addition, strong perceived safety created the opportunity to leave the hospital earlier due to the feeling of safe care [12, 41], while low perceived safety could lead to a loss of trust in healthcare and politics due to non-functioning technology [57]. Furthermore, economic outcomes could occur, such as by allowing a more efficient allocation of resources through the increased use of DTs [7, 12], thus saving costs [6]. Despite the positive outcomes of perceived safety, it is important to be aware of negative outcomes such as the risk of false perceived safety, which can lead to a lack of attention [7]. Alternatively, increased DT use can encourage avoidance strategies in the face of the unknown, hindering personal development [55].

## Influencing factors and needs in the context of feeling safe and digital technologies in healthcare

**Fig. 2:**
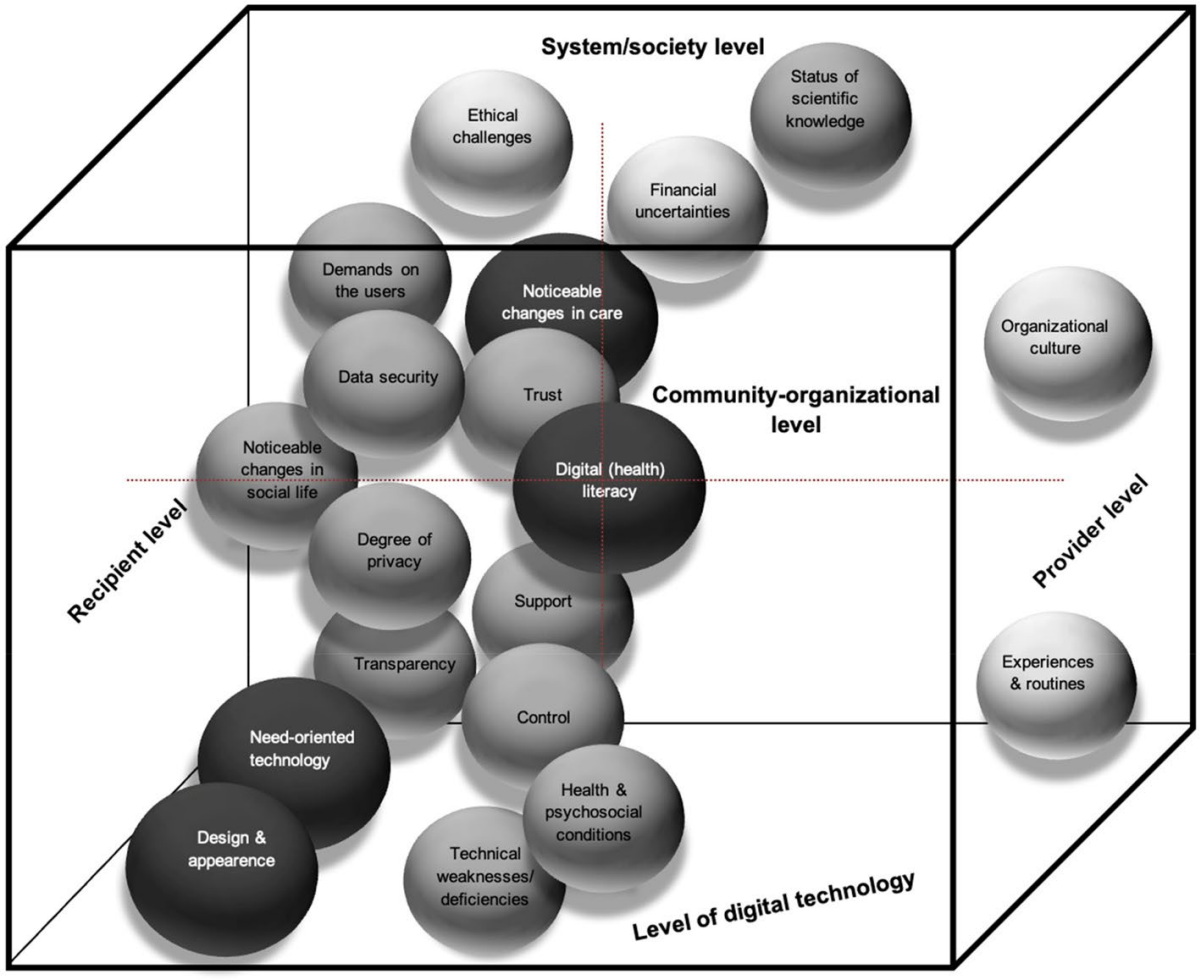
Core dimensions of IFs in the context of perceived safety and DTs in healthcare. Legend: larger black spheres = mostly addressed CDIFs; smaller lighter spheres = rarely addressed CDIF; bottom layer = level of digital technology; left layer = healthcare recipient level; right layer = healthcare provider level; upper layer = system/society level; and rear layer = community-organizational level)

Nineteen CDIFs and needs related to feeling safe and DTs (see Fig. 2) were developed based on 127 main and 222 sub-categories (76.59% of the subcategories were related to emotional safety, and 23.41% were related to psychological safety) (see Additional file 3). Each CDIF was multifaceted in its dimensions and addressed one or several levels: the microlevel (healthcare recipient/provider and DT), mesolevel (community/organizational), and macrolevel (system/society).

Most of the CDIFs (*n* = 15) were primarily at the healthcare recipient level, and the DT, community-organizational, and system/society levels were considered nearly equally. Only two CDIFs (‘organizational culture’; and ‘experiences and routines’) were on the healthcare provider and considered the organizational level and individual experiences. Several CDIFs at the healthcare recipient/provider level were directly related to previous experience and user characteristics, as well as to the DT. A large number of CDIFs were related to the community-organizational level, as they represent community-related issues or effects before and after the use of DTs. A few CDIFs could be assigned to the system-society level, which involves fundamental societal uncertainties that may have an impact on the perceived safety before the introduction of DTs.

Most of the CDIFs were also related to needs that were described for both healthcare recipients and providers, e.g., receiving support [52] (see Additional file 4). Not receiving necessary support negatively influences perceived safety [48]. Some CDIFs were not addressed such as ‘trust’, ‘data security’, ‘transparency’, ‘demands on the users’, ‘financial uncertainties’, and ‘organizational culture’. Generally, the needs were categorized into three main areas: needs for the stakeholders themselves (e.g., healthcare recipients expressed the desire for anonymity or autonomy in decision-making [36, 55]); needs for DTs (e.g., DTs should have different features depending on individual needs, better monitoring [48] or special features for emergency situations [54]); and needs for the environment (e.g., creating the right conditions for DT implementation [38, 42, 47], including organizational and political support [42]).

### Core dimensions of influencing factors, most frequently addressed by publications

Nineteen CDIFs were identified by publications at varying frequencies. They could be divided into three groups: ‘mostly addressed’ *n* = 16–24 (50–75%), ‘moderately addressed’ *n* = 3–14 (9–44%), and ‘rarely addressed’ *n* = 1–2 (3–6%).

Four of the 19 CDIFs, ‘noticeable changes in care’, ‘digital (health) literacy’, ‘design and appearance’, and ‘need-oriented technology’, were mostly addressed by publications and had both positive and negative influences on feeling safe. The most addressed CDIF, ‘noticeable changes in care’, covered the perception of changes in people’s care and its association with perceived safety on several levels. This reflected perceived health improvements, such as more efficient access to care [6, 7, 40] or resolved communication problems by DTs [3, 41, 56]. In contrast, a fear of deterioration by DTs [36, 38] or expected reduced access for certain patient groups because of an inability to use DTs could lead to a loss of perceived safety [38, 42].

The second most frequently addressed CDIF, ‘digital (health) literacy’, reflected nearly all levels of healthcare recipients and providers and DTs at the community-organizational, system, and society levels. It contained several aspects of DT affinity [39] and knowledge [43], such as the manner in which (needs-based) information is communicated [43] and dealing with DTs are trained [60]. In this context, high DT-related requirements in society seem to be relevant for people who are particularly unfamiliar with technical devices [9, 52], whereas the limited capabilities of healthcare recipients or providers can be a barrier to perceived safety [38, 42].

The third most frequently addressed CDIF, ‘design and appearance’ (DT/organizational level), contained predominantly positive IFs of feeling safe, e.g., the empowerment of healthcare recipients working with DTs [12, 37, 43]. Notably, the human appearance of robots was viewed differently. For some healthcare recipients, a high degree of humanity can lead to increased perceived safety; for others, the opposite is true [3]. Additionally, the visibility or invisibility of the DTs can lead to an opposing perceived safety [3, 6, 44]. Different views on feeling safe were also found regarding the fourth most often addressed CDIF, ‘need-oriented technology’, which stresses the importance of DTs that are adapted to the needs, expectations, and values of users [6, 9, 54]. It was emphasized that it is essential to involve the users of DTs as co-designers [3] to ensure user-specific DTs [2, 43]. The relevance of adapting DTs to an individual’s disease needs was stressed to avoid reduced safety feeling [46].

### Core dimensions of influencing factors focusing on the healthcare recipient and other levels

The CDIF ‘control’ (recipient and organizational level) played a crucial role from healthcare recipients’ point of view, as the opportunity to control the DT to stop it [55], the control over one’s own data [3, 7, 45] or the supervision of the DT by healthcare professionals [3] are mentioned positively. Restrictions on data access rights for healthcare providers resulted in minor control of health data and hence lower perceived safety at the recipient and system levels [45]. Similarly, the CDIFs’ ‘transparency’ (e.g., data processing [3, 9] and data transparency [7, 45, 46]) and ‘degree of privacy’ (e.g., protection of privacy [48] and a risk–benefit analysis [54]) were associated with the healthcare recipient and organizational levels. ‘Transparency’ was found to hinder perceived safety, mainly because unclear data transfer processes caused confusion [7, 40]. The ‘degree of privacy’ was important for healthcare recipients and for social debates [47]. The CDIFs ‘data security’ and ‘demands on the users’ were both identified as inhibiting perceived safety since fundamental societal concerns about data security were expressed [9, 41, 45]. Users experience high political and societal demands because they are expected to know how to use DTs [9]. Overall, from the point of view of healthcare recipients, DT develops rapidly, which can lead to a fear of anticipated excessive demands and negative feelings such as stress or anxiety about using new DTs [52], especially among older people [9].

### Core dimensions of influencing factors, emphasizing healthcare provider, and other levels

Four CDIFs at the healthcare provider level have rarely been addressed in publications but have had an impact on the perceived safety. ‘Financial uncertainties’ are uncertainties about the financial remuneration of digital services via ‘providers’ [36, 42]; ‘status of scientific knowledge’ is related to unresolved scientific questions that can create ambiguity for healthcare recipients in therapies [52, 60] addressed at the system/society level; ‘organizational culture’ refers to healthcare providers feeling safer and testing new DTs in a positive organizational culture where initial mistakes regarding DT use are tolerated [53]; and ‘experiences and routines’, make healthcare providers feel more confident in using DTs at an individual level and allow them to develop a safety feeling due to exercises and routines [38, 43].

### Relationships among the core dimensions of influencing factors

Although only a few relationships among the CDIFs were explicitly mentioned, this did not necessarily mean that none existed. For example, ‘need-oriented technology’ is related to ‘health and psychosocial conditions’. The physical conditions and illnesses of healthcare recipients were described as having an impact on perceived safety regarding DT use [3, 43, 50] for both healthcare recipients and providers [42]. Hence, considering needs is also related to considering health status and the appropriate selection of DTs [2, 43]. ‘Support’ and ‘trust’ could both be similar ‘digital (health) literacy’ considering the healthcare recipient and provider levels nearly evenly. ‘Support’ (e.g., the presence of healthcare providers during DT use) [3, 43] and the opportunity to receive efficient support if required [6, 9, 44] were more directed to the healthcare provider level, suggesting that a lack of support for technical problems can restrict the perceived safety [48]. The CDIF ‘trust’ included, for example, increased trust by DTs [3, 40], the perception of DTs as ‘cold and distant’ [2], and limited general openness of healthcare recipients towards DTs [38].

‘Noticeable changes in social life’ and ‘noticeable changes in care’ both focus on perceived changes due to DT use. Healthcare recipients often perceive a change in their social life and environment due to the use of DTs positively affects their feeling of safety [6] because DTs enable individuals to stay at home [40, 47, 60] or create a safe space at home [40, 55, 57]. This point was made only by the healthcare recipients and their relatives. In contrast, the CDIF ‘noticeable changes in care’ were also mentioned by healthcare providers, such as improved or faster care via DTs [6, 41, 42]. Inhibiting factors were also noted by healthcare providers and recipients, such as anxiety about the loss of human interaction [39, 43, 60]. This can be interpreted as a perceived deterioration of care and could lead to an inhibited safety feeling [43].

### Core dimensions of influencing factors related to DTs

All CDIFs covered by needs were related to one or more of the eleven types of DTs, and none were addressed by all types, such as robotics in healthcare (*n* = 3) [3, 39, 59]; telehealth (remote healthcare by telecommunication, e.g., telemedicine, telenursing, telecare) (*n* = 10) [12, 38, 40, 42–44, 47, 51, 56, 60]; e-Health general (e.g., digital data usage, communication, digitally led healthcare, experiences with eHealth) (*n* = 7) [7, 9, 45, 50, 53, 57, 58]; telemonitoring (monitoring process that allows a remote interpretation of the necessary data) (*n* = 6) [41, 44, 46, 48, 52, 54]; digital apps to facilitate health self-management (*n* = 1) [49]; camera surveillance (*n* = 1) [6]; an internet-based group platform that facilitates exchange among affected people) (*n* = 1) [36]; digital personal health information management (*n* = 1) [37]; digital medicine dispenser (*n* = 1) [2]; online counselling (*n* = 1) [55]; and participant simulation programme (*n* = 1) [35]. One study [44] combined two different DTs (telecare and telemonitoring).

In total, 244 allocations of DT types to the main categories were applied, with the overall result that telehealth was most often explored and addressed in terms of feeling safe (28.28%), followed by telemonitoring (20.08%), eHealth in general (16.39%) and robotics (13.52%). All the other DTs were addressed less frequently (< 5.33% per DT). The DT ‘Participation Simulation Programme’ has been addressed very little and could be assigned to only one CDIF (‘support’). ‘Noticeable changes in care’ was addressed by eight different DTs, as was ‘digital (health) literacy’. However, with respect to the nine DTs, ‘need-oriented technology’ had a high diversity of DTs but fewer different literature sources. In contrast, ‘organizational culture’ was the least represented (only one study), closely followed by ‘experiences and routines’ (two), both of which were addressed by one DT. The exclusively negative CDIF ‘technical weaknesses/deficiencies’, mentioned by both healthcare recipients and providers, reflected the adverse influence on perceived safety in the case of missing functionality of the DT [41, 42, 44].

### Ethical, psychosocial, economic, political, and legal perspectives in the context of feeling safe and DTs in healthcare

A few findings could be derived from ethical, economic, psychosocial, and political perspectives, and no statements could be identified from legal perspectives (see Additional file 5). ‘Ethical challenges’ included fundamental ethical questions before DT use [9, 48] or the appropriate level of video recording that is ethically acceptable (system level) [48]. It was assumed that if these issues remain unresolved, they can become an inhibiting factor [9, 48]. Ethical competence was important [6], and the physical and psychological integrity of healthcare providers was not compromised during DT use. Furthermore, ethical principles should remain valid after DT implementation [58]. The benefit of the DT needs to be weighed against potential disadvantages and ethical outcomes [2]. Ethical concerns emerged in relation to the over-humanization of DTs, with a need to address the appropriate extent to which a humanizing approach to robots in care relationships is advisable or contraindicated, as this remains a research gap [59]. Privacy as an ethical issue came to the fore, although safety concerns were prioritized [48]. Finally, being confronted with DT situations for which one was unprepared was considered unethical, highlighting the importance of adequate preparation and training when implementing DTs to ensure ethical safe practices in healthcare [9].

From a psychosocial perspective, enhanced DT social networks allow individuals to connect with others and foster meaningful relationships [6]. However, patients might encounter greater safety risks while aiming to remain at home, underscoring the importance of thoroughly assessing potential hazards [37]. In addition, DTs enable communication with other people, facilitating emotional expression and support across a spectrum of emotions [59]. Understanding the related psychosocial dimensions, facilitates connectedness, emotional well-being, and safety for people seeking care and support [6].

The economic perspective has highlighted the potential for significant economic and practical benefits [6] that are associated with the use of DTs, including economic and time savings [42, 47]. However, it was stressed that the non-adoption of DTs can occur due to perceived costs [6]. The main barriers to acquiring digital literacy skills are often associated with training and education costs [47]. Efforts should be made to reduce purchase costs and remove barriers to the use of telehealth services for healthcare recipients’ personal devices [12]. When financial concerns were alleviated, new DTs could be developed and tested without excessive concern regarding their acceptance, as the potential economic benefits outweigh other considerations [6].

The political perspective encompasses sociotechnical complexities and the need to address them to ensure successful implementation [46]. Policy-makers play a crucial role in this process as they are asked to think about how to manage DTs at both the individual and societal levels [57] considering the needs and preferences of different stakeholders, including healthcare recipients, providers, and society [48, 57].

## Discussion

Our scoping review aimed to analyze the literature on the needs and influencing factors in the context of emotional and psychological safety in relation to healthcare digitalization and DT use to gain a multiperspective and comprehensive understanding. Despite an extensive and sensitive search, only a few studies could be identified that primarily focused on exploring the perceived safety of DTs in healthcare. The key findings highlight that perceived safety is a complex and multidimensional phenomenon that is influenced by factors at the DT, individual, community-organizational, and system-society levels.

Investigations of emotional safety are in line with current trends and are important in healthcare [5]. Although psychological safety has been a focus of research for several years [21] and the role of healthcare providers in responsible digital health is becoming increasingly important [8], we observed that it currently plays a minor role in the context of digitalization. This is underlined by a meta-analysis in 2014 that dealt extensively with psychological safety in everyday working life. However, digitalization in healthcare did not play a role in this research [21].

Several CDIFs were addressed by both healthcare recipients and providers. However, the overall focus was on healthcare recipients. From that perspective, research gaps and ethical/financial context conditions were identified that seem to be of fundamental relevance at the system level. Their clarification seems essential, as those gaps and unexplored conditions can contribute to perceptions of being unsafe [42, 48, 52]. Society should acknowledge the importance of comprehending the capabilities and limitations of DTs to avoid a pervasive sense of feeling unsafe [43]. However, while a few CDIFs were addressed at the system-society level, most were focused on the community-organizational level. This might show that CDIFs are strongly perceived by the community and reflect high social relevance, which is comparable to the results of van Hoof et al. [61], who found in a different context that community and social participation were important aspects of feeling safe.

We found that digital (health) literacy was central, with an impact on all levels, and seemed to be influenced by all levels; for example, digital (health) literacy is strongly dependent on the health and psychosocial conditions of healthcare recipients [3]. These findings align with the results of Zhou et al. [62], who stressed that digital (health) literacy is crucial for the demand for health services among older people in the context of digital transformation. We have found that the implementation of DTs in the health sector requires a holistic approach, as the perceived safety of dealing with DTs is relevant at different contextual levels. This aligns with Pfadenhauer's description that complex interventions require ongoing analysis and development of tailored interventions and implementation strategies [63]. Akalin et al. [11] investigated the IFs of the perceived safety of social robots for young adults in human robot interaction. The six described IFs (comfort, predictability, sense of control, transparency, trust, experience, and familiarity) [11] were similar to ours.

Only a few user needs were identified. Most of the needs presented coincided with the facilitating factors, suggesting that needs play an active role in influencing the behavior, decisions, or attitudes of the individuals involved and revealing common ground in the quest for enhanced safety [48]. Not all CDIFs were addressed by needs, which may underscore the existence of pivotal factors that play a substantial role in feeling safe but might not be immediately expressed as explicit needs.

In healthcare, a human-centered approach proves to be particularly important in digitalization, as emphasized by the German government’s Data Ethics Commission [58]. Hence, it is not just a matter of developing DTs to what is technically feasible [58]. Very few needs were identified from a healthcare provider perspective, reflecting the previous focus on other security aspects, such as data protection, data security, and information security, as described by Okhrimenko et al. [4].

The individual perceived safety by all users seems to play a decisive role in the outcomes of feeling safe. For example, subjectively perceived changes can lead to different individual assessments of outcomes [3]. A change in the perceived safety primarily leads to new attitudes and influenced feelings towards DTs, which in turn seems to influence user behavior [44]. This user behaviour can in turn have far-reaching effects on health economic and healthcare system aspects [6, 7, 12]. A similar result has already been discussed in implementation research by Lewis et al. [64]. The interactions among perceptions of safety, attitudes, use behavior and the resulting outcomes illustrate the far-reaching importance of feeling safe in the implementation and use of DT; for example, Nyholm et al. [3] already described the safety feeling as the cornerstone of future healthcare robotics. Lyndon et al. [5] extend this by describing that feeling safe is a core component of patient safety and should play a role in any future implementation as well as patient safety measurement.

The implementation of DTs can influence participants’ perceived safety in both positive and negative ways [18, 46]; for example, they may experience fewer worries when using DTs, resulting in greater perceived safety [44]; alternatively, they may experience a sense of failure when using DTs and not being able to cope with the challenges of DTs, resulting in low perceived safety [9]. These two influences underscore the complexity of integrating new DTs into healthcare, which is expected to lead to behavioral changes related to skepticism [25]. Initial research suggests that this skepticism or uncertainty in use may also be relevant to reduced perceived safety [11]. Among other things, the individual preconditions of the users and the corresponding framework conditions seem to be relevant [43]. Notably, strong perceived safety does not necessarily always lead to positive outcomes; rather, an overly strong perceived safety can also lead to potential risks or problems being neglected [7, 55]. Previous studies have already discussed the complexities of this implementation [8] and the difficulties of general implementation in healthcare [23]. It is important to carefully consider both the positive and potentially negative aspects to ensure successful implementation [58, 59].

Discussion of the impact of DTs on perceived safety in healthcare raises important questions, particularly in relation to the ethical and legal framework [48]. While ethical guidelines exist, there is currently a lack of legal guidance on the management and impact of DTs on perceived safety [59], supported by the fact that no legal statements have been found in relation to perceived safety. This gap can make people feel unsafe and highlights the need to develop regulatory measures to ensure the protection of users when working with DTs [59]. It is important that policymakers create appropriate frameworks to regulate the use of DTs and ensure that ethical standards and privacy principles can be met [58]. Moreover, economic factors play a role, as individual decisions must be made as to whether the use of DTs is financially rewarding [36, 42], as Raimo et al. [65] described economic factors as one of the drivers of digital transformation. The trade-offs among investment costs, long-term benefits, and impacts on perceived safety can be complex and require informed decision-making, which can also be guided by the fact that healthcare providers have the financial certainty that they will be adequately reimbursed for the use of DTs. As James et al. [42] describe how during the COVID-19 pandemic, caregivers in Australia could not bill for their telecare services because there was no system in place to do so and, as a consequence, did not feel safe.

## Limitations

This scoping review has considered scientific standards. According to Krippendorff [33], an independent content analysis of the literature might minimize the risk of bias. Here, the coding was conducted by one of the authors; however, coding supervision was conducted by another author. Although a very sensitive search strategy was used and grey literature was included, due to the inclusion criteria and the search period, it might be that we did not identify all relevant publications. In line with PRISMA-ScR [26], we did not perform a critical appraisal of the included publications.

The quality of the definitions and descriptions of perceived safety differed among the publications, which could have resulted in interpretation bias. The CDIFs were multidimensional because they considered various types of DTs classified according to their type. However, due to the lack of scientific publications, this information can be used only to a limited extent. More types of DTs should be developed to gain a deeper understanding of how CDIFs affect perceived safety.

## Conclusion

Feeling safe in the context of DTs in healthcare is a complex and multifaceted phenomenon. Therefore, this scoping review underlines the need for a holistic approach to digital transformation, as it shows that IFs and needs require the multilevel development of interventions and implementation strategies that consider all stakeholders to improve perceived safety. In particular, the promotion of the digital (health) literacy of healthcare providers and recipients can be highlighted as a key factor for perceived safety. In addition, the integration of DTs in healthcare can have both positive and negative effects on feeling safe, highlighting the need for balanced, individualized considerations of their implementation. While emotional safety is gaining attention, the psychological safety related to digitalization in healthcare remains relatively unexplored in research. Due to the complexity of these issues, the different stakeholders involved, and the high importance of perceived safety, especially among healthcare providers, feeling safe can contribute to patient safety, but additional research is needed.

### Supplementary Information

**Additional file 1. **Data searching strategies.

**Additional file 2. **Outcomes of a strong perceived safety.

**Additional file 3. **Outcomes of a low perceived safety.

**Additional file 4. **Influencing factors and needs in the context of perceived safety and digital technologies in healthcare.

**Additional file 5. **Perspectives on emotional or psychological safety (explicit mentioned in included literature).

## Data Availability

All supplementary data can be provided by the corresponding author upon request.

## Abbreviations

BMBF: Federal Ministry of Education and Research
CDIFs: Core dimensions of influencing factors
DTs: Digital technologies
IFs: Influencing factors
JBI: Joanna Briggs Institute
MeSH: Medicine Subject Headings
PRESS: Peer Review of Electronic Search Strategies checklist
PICo: Population, the Phenomena of Interest, and the Context
PRISMA-ScR: Preferred Items for Reporting on Systematic Reviews and Meta Analyses-Extension for Scoping Reviews
SteTiG: Emotional safety as a condition for success in the digital transformation
