# Supplementary material for: Feeling safe in the context of digitalization in healthcare: a scoping review
Source: Syst Rev. 2024 Feb 8;13:62. doi: 10.1186/s13643-024-02465-9 (PMC10851492; doi:10.1186/s13643-024-02465-9)
Supplement: Supplementary file 1 — Additional file 1. Data searching strategies. [file 13643_2024_2465_MOESM1_ESM.docx]

**Additional file 1**

**Search Strategy for MEDLINE (via PubMed)**

| Research period: 01 October 2017 to 30 September 2022  Date of research: 26.10.2022 | | |
| --- | --- | --- |
| Step of Search | Hits | Search Terms |
| 1 | 725,360 | "telemedicine"[MeSH Terms] OR "robotics"[MeSH Terms] OR "robotic surgical procedures"[MeSH Terms] OR "artificial intelligence"[MeSH Terms] OR "machine learning"[MeSH Terms] OR "telenursing"[MeSH Terms] OR "electronic health records"[MeSH Terms] OR "mobile applications"[MeSH Terms] OR "medical records"[MeSH Terms] OR "remote consultation"[MeSH Terms] OR "e-Health"[Title/Abstract] OR "ehealth"[Title/Abstract] OR "m-health"[Title/Abstract] OR "mhealth"[Title/Abstract] OR "mobile health"[Title/Abstract] OR "electronic health"[Title/Abstract] OR "telehealth"[Title/Abstract] OR "telematic*"[Title/Abstract] OR "telemedicine"[Title/Abstract] OR "telenurs*"[Title/Abstract] OR "teletherap*"[Title/Abstract] OR "telemonitor*"[Title/Abstract] OR "artificial intelligence"[Title/Abstract] OR "ai"[Title/Abstract] OR "robotic*"[Title/Abstract] OR "digital health"[Title/Abstract] OR "digital public health"[Title/Abstract] OR "electronic medical record*"[Title/Abstract] OR "emr"[Title/Abstract] OR "tele-education*"[Title/Abstract] OR "Teleeducation*"[Title/Abstract] OR "telehomecare*"[Title/Abstract] OR "teleconsultation*"[Title/Abstract] OR "virtual*"[Title/Abstract] OR "remote"[Title/Abstract] |
| 2 | 1,687,548 | "safety"[MeSH Terms] OR "patient harm"[MeSH Terms] OR "patient safety"[MeSH Terms] OR "safety management"[MeSH Terms] OR "risk management"[MeSH Terms] OR "safe"[Title/Abstract] OR "safeties"[Title/Abstract] OR "safety"[Title/Abstract] OR "secure"[Title/Abstract] OR "secureness"[Title/Abstract] OR "safeness"[Title/Abstract] OR "harm"[Title/Abstract] OR "harms"[Title/Abstract] OR "threatened"[Title/Abstract] OR "threats"[Title/Abstract] OR "threat"[Title/Abstract] OR "threateningly"[Title/Abstract] OR "threatens"[Title/Abstract] OR "threatful"[Title/Abstract] OR "uncertain"[Title/Abstract] OR "uncertainty"[Title/Abstract] OR "unsure"[Title/Abstract] OR "insecure"[Title/Abstract] OR "unsafe"[Title/Abstract] |
| 3 | 629,382 | "emotions"[MeSH Terms] OR "emotions"[Title/Abstract] OR "emotion"[Title/Abstract] OR "socio emotion"[Title/Abstract] OR "feeling"[Title/Abstract] OR "feelings"[Title/Abstract] OR "feel"[Title/Abstract] OR "feel*"[Title/Abstract] OR "emotional"[Title/Abstract] OR "socio-emotional"[Title/Abstract] OR "socioemotional"[Title/Abstract] |
| 4 | 2,155,528 | "psychological distress"[MeSH Terms] OR "psych*"[Title/Abstract] OR "experience"[Title/Abstract] OR "experienced"[Title/Abstract] OR "experiences"[Title/Abstract] |
| 5 | 2,097 | #1 AND #2 AND #3 |
| 6 | 12,412 | #1 AND #2 AND #4 |
| 7 | 13,418 | #5 OR #6 |
| 8 | 10,983 | #7 AND medline[sb] |
| 9 | 5,352 | #8 AND (“2017/10/01”[Date - Publication] : “2022/09/30”[Date - Publication]) |

**Search Strategy for CINAHL (via EBSCO)**

| Research period: 01 October 2017 to 30 September 2022  Date of research: 18.10.2022 | | |
| --- | --- | --- |
| Step of Search | Hits | Search Terms |
| 1 | 181,279 | (MH telehealth OR MH telemedicine OR MH "remoteconsultation" OR MH telepathology OR MH teleradiology OR MH telerehabilitation OR MH teledentistry OR MH telepsychiatry OR MH telenursing OR MH "mobile healthunits" OR MH "electronic healthrecords" OR MH "digital health" OR MH "wearable sensors" OR MH "ambulatorycare nursing" OR MH "artificial intelligence" OR MH "roboticsurgical procedures" OR MH "mobileapplications" OR MH "remote consultation")OR TI "e-health" OR AB "e-health" OR TI ehealth OR AB ehealth OR TI "m-health" OR AB "m-health" OR TI mhealth OR AB mhealth OR TI "mobile health" OR AB "mobile health" OR TI "electronic health" OR AB "electronic health" OR TI telehealth OR AB telehealth OR TI telematic* OR AB telematic* OR TI telemedicine OR AB telemedicine OR TI telenurs* OR AB telenurs* OR TI teletherap* OR AB teletherap* OR TI telemonitor* OR AB telemonitor* OR TI "artificial intelligence" OR AB "artificialintelligence" OR TI ai OR AB ai OR TI robotic* OR AB robotic* OR TI "digitalhealth" OR AB "digitalhealth" OR TI "digitalpublic health" OR AB"digital public health" OR TI "electronicmedical record" OR AB "electronic medicalrecord" OR TI emr OR AB emr OR TI "tele-education" OR AB "tele-education" OR TI teleeducation OR AB teleeducation OR TI telehomecare OR AB telehomecare OR TI teleconsultation OR AB teleconsultation OR TI virtual* OR AB virtual* OR TI remote OR AB remote |
| 2 | 570,663 | (MH safety+) OR (MH„patient safety+“) OR MH „risk assessment“ OR (MH „riskmanagement+“) OR TI safe OR AB safe OR TI safeties OR AB safeties OR TI safety OR AB safety OR TI secure OR AB secure OR TI secureness OR AB secureness OR TI safeness OR AB safeness OR TI harm OR AB harm OR TI harms OR AB harms OR TI threatened OR AB threatened OR TI threats OR AB threats OR TI threat OR AB threat OR TI threateningly OR AB threateningly OR TI threatens OR AB threatens OR TI threatful OR AB threatful OR TI uncertain OR AB uncertain OR TI uncertainty OR AB uncertainty OR TI unsure OR AB unsure OR TI insecur* OR AB insecur* OR TI unsaf* OR AB unsaf* |
| 3 | 293,035 | (MH emotions+) OR TI emotions OR AB emotions OR TI emotion OR AB emotion OR TI feeling OR AB feeling OR TI feelings OR AB feelings OR TI feel OR AB feel OR TI feel*OR AB feel* OR TI emotional OR AB emotional OR TI "socio-emotional" OR AB "socio-emotional" OR TI „socioemotional“ OR AB „socio emotional“ OR TI socioemotional OR AB socioemotional |
| 4 | 805,007 | MH „psychologicalsafety“ OR MH „Human needs(psychology)“ OR TI psych* OR AB psych*OR TI experience OR AB experience OR TI experienced OR AB experienced OR TI experiences OR AB experiences |
| 5 | 930 | S1 AND S2 AND S3 |
| 6 | 4,064 | S1 AND S2 AND S4 |
| 7 | 5,001 | S5 OR S6 |
| 8 | 2,921 | S7 (Published Date: 20171001-20220931) |

**Search strategy for (PsycINFO via Ovid)**

| Research period: 01 October 2017 to 30 September 2022  Date of research: 25.10.2022 | | |
| --- | --- | --- |
| Step of Search | Hits | Search Terms |
| 1 | 104,130 | Electronic Health Services/ or Telemedicine/ or Computer assisted therapy/ or Online therapy/ or Teleconferencing/ or Teleconsultation/ or Telepsychiatry/ or Telepsychology/ or Telerehabilitation/ or Digital interventions/ or Mobile health/ or Computer mediated communication/ or Machine learning/ or Social robotics/ or e-Health.ti. or e-Health.ab. or ehealth.ti. or ehealth.ab. or m-health.ti. or m-health.ab. or mhealth.ti. or mhealth.ab. or mobile health.ti. or mobile health.ab. or electronic health.ti. or electronic health.ab. or telehealth.ti. or telehealth.ab. or telematic$.ti. or telematic$.ab. or telemedicine.ti. or telemedicine.ab. or telenurs$.ti. or telenurs$.ab. or teletherapy$.ti. or teletherap$.ab. or telemonitor$.ti. or telemonitor$.ab. or artificial intelligence.ti. or artificial intelligence.ab. or ai.ti. or ai.ab. or robotic$.ti. or robotic$.ab. or digital health.ti. or digital health.ab. or digital public health.ti. or digital public health.ab. or electronic medical record$.ti. or electronic medical record$.ab. or emr.ti. or emr.ab. or tele-education$.ti. or tele-education$.ab. or teleeducation$.ti. or teleeducation$.ab. or telehomecare$.ti. or telehomecare$.ab. or teleconsultation$.ti. or teleconsultation$.ab. or virtual$.ti. or virtual$.ab. or remote.ti. or remote.ab. |
| 2 | 274,456 | Safety/ or patient safety/ or threat/ or risk management/ or uncertainty/ or Safe.ti. or safe.ab. or safeties.ti. or safeties.ab. or safety.ti. or safety.ab. or secure.ti. or secure.ab. or secureness.ti. or secureness.ab. or safeness.ti. or safeness.ab. or harm.ti. or harm.ab. or harms.ti. or harms.ab. or threatened.ti. or threatened.ab. or threats.ti. or threats.ab. or threat.ti. or threat.ab. or threateningly.ti. or threateningly.ab. or threatens.ti. or threatens.ab. or threatful.ti. or threatful.ab. or uncertain.ti. or uncertain.ab. or uncertainty.ti. or uncertainty.ab. or unsure.ti. or unsure.ab. or insecur$.ti. or insecur$.ab. or unsaf$.ti. or unsaf$.ab. |
| 3 | 467,471 | Emotions/ or emotions.ti. or emotions.ab. or emotion.ti. or emotion.ab. or feeling.ti. or feeling.ab. or feelings.ti. or feelings.ab. or feel$.ti. or feel$.ab. or emotional.ti. or emotional.ab. or socio-emotional.ti. or socio emotional.ab. or socioemotional.ti. or socioemotional.ab. |
| 4 | 1,769,618 | Psych$.ti. or psych$.ab. or experience.ti. or experience.ab. or experienced.ti. or experienced.ab. or experiences.ti. or experiences.ab. |
| 5 | 831 | 1 and 2 and 3 |
| 6 | 2,670 | 1 and 2 and 4 |
| 7 | 3,000 | 5 or 6 |
| 8 | 1,543 | limit 7 to up=20171001-20220930 |

**Search Strategy for Google Scholar**

| Research period: 2017-2023  Date of research: 25.10.2022 | | |
| --- | --- | --- |
| Step of Search | Hits | Search Terms |
| 1 | 1250 | “emotional safety” AND “~e-health” OR “~m-health” OR “~mobile health” OR “~electronic health” OR “~telehealth” OR “~telemedicine” OR “~telenurse” OR “~teletherapy” OR “~artificial intelligence” OR “~robotic” OR “~digital health” |
| 2 | 850 | Limit to 2017-2023 |

| Research period: 2017-2023  Date of research: 25.10.2022 | | |
| --- | --- | --- |
| Step of Search | Hits | Search Terms |
| 1 | 6500 | “psychological safety” AND “~e-health” OR “~m-health” OR “~mobile health” OR “~electronic health” OR “~telehealth” OR “~telemedicine” OR “~telenurse” OR “~teletherapy” OR “~artificial intelligence” OR “~robotic” OR “~digital health” |
| 2 | 5000 | Limit to 2017-2023 |

| Research period: 2017-2023  Date of research: 25.10.2022 | | |
| --- | --- | --- |
| Step of Search | Hits | Search Terms |
| 1 | 107 | "psychologische Sicherheit" AND „Digitalisierung im Gesundheitswesen“ OR „~telenurse“ OR „~teletherapie“ OR „~künstliche Intelligenz“ OR „~Robotik“ OR „~telemedizin“ OR „~mobile health“ |
| 2 | 92 | Limit to 2017-2023 |

| Research period: 2017-2023  Date of research: 25.10.2022 | | |
| --- | --- | --- |
| Step of Search | Hits | Search Terms |
| 1 | 52 | "emotionale Sicherheit" AND „Digitalisierung im Gesundheitswesen“ OR „~telenurse“ OR „~teletherapie“ OR „~künstliche Intelligenz“ OR „~Robotik“ OR „~telemedizin“ OR „~mobile health“ |
| 2 | 34 | Limit to 2017-2023 |
