# Supplementary material for: Feeling safe in the context of digitalization in healthcare: a scoping review
Source: Syst Rev. 2024 Feb 8;13:62. doi: 10.1186/s13643-024-02465-9 (PMC10851492; doi:10.1186/s13643-024-02465-9)
Supplement: Supplementary file 2 — Additional file 2. Outcomes of a strong perceived safety. [file 13643_2024_2465_MOESM2_ESM.docx]

**Additional file 2**

| **Additional file 2: Outcomes of a strong perceived safety** | | | | | | | | | | | | | | |
| --- | --- | --- | --- | --- | --- | --- | --- | --- | --- | --- | --- | --- | --- | --- |
| **Outcomes of a strong perceived safety** | | **Context of Outcomes** | **Source** | **Digital technologies** | | | | | | | | | | |
| **Main category** | **Sub category** |  |  | **1** | **2** | **3** | **4** | **5** | **6** | **7** | **8** | **9** | **10** | **11** |
| **Healthcare recipients** | | | | | | | | | | | | | | |
| **Positive feelings/perceptions of healthcare recipients due to DTs** | **Less worries when using DT** | Telecare could lead to feel extra safe and less worried | [44] |  | X |  | X |  |  |  |  |  |  |  |
|  | **Sense of dignity by using DT** | DTs help to improve persons’ sense of dignity and have an impact on the perceived safety | [9] |  |  | X |  |  |  |  |  |  |  |  |
|  | **Perception of personal control through appropriate help using DT** | Suitable help to use new technology and support through eHealth can provide a perceived safety and thereby improve older persons’ perception of personal control | [9] |  |  | X |  |  |  |  |  |  |  |  |
|  | **Well-being of older people by using DT** | Improve older adults’ well-being | [9] |  |  | X |  |  |  |  |  |  |  |  |
|  | **Openness, improved problem discussion & improved mental effects by using DT** | DT can promote the patients’ openness to communication with other people, train and keep vital the range of their emotional opportunities, strengthen their perceived safety, and produce other mental effects that are also beneficial | [59] | X |  |  |  |  |  |  |  |  |  |  |
|  | **Feelings of higher participation by using DT** | Feeling of being better or more involved in one’s own care | [7] |  |  | X |  |  |  |  |  |  |  |  |
|  | **Feeling of being acknowledged and monitored by using DT** | DT could help healthcare recipients feel recognised and monitored in a positive and safe way | [49] |  |  |  |  | X |  |  |  |  |  |  |
|  | **Motivation to use DTs to remain in their own home** | Improved safety can motivate the use of DTs to support ageing in place | [6] |  |  |  |  |  | X |  |  |  |  |  |
|  |  | Due to the possibility to remain in their current dwellings | [44] |  | X |  | X |  |  |  |  |  |  |  |
| **Using DTs leads to further opportunities of healthcare recipients** | **Opportunity for earlier hospital discharge when using DT** | Possibility to go home and still have the support due to the DT was described as positive | [41] |  |  |  | X |  |  |  |  |  |  |  |
|  |  | The DT made it safer to leave the hospital knowing how easily you could reach the nurses | [12] |  | X |  |  |  |  |  |  |  |  |  |
|  | **Opportunity to take advantage of further therapies by using DT** | A high level of safety in the online setting due to the distance can act as a starting point and end in a face to face consultation | [55] |  |  |  |  |  |  |  |  |  | X |  |
|  | **Opportunity to age in place by using DT** | Older patients can age in place | [6] |  |  |  |  |  | X |  |  |  |  |  |
|  |  | Opportunity to remain in their current dwellings | [44] |  | X |  | X |  |  |  |  |  |  |  |
|  | **The possibility of choosing DT over human healthcare** | Camera surveillance was experienced as giving an increased safety feeling at night than surveillance by third parties | [44] |  | X |  | X |  |  |  |  |  |  |  |
| **Benefits for healthcare recipients due to DTs in healthcare & healthcare management** | **Increased independence by using DT** | DT enabled increased independence in many cases | [44] |  | X |  | X |  |  |  |  |  |  |  |
|  | **Increased predictability in daily life by using DT** | Increased predictability in daily life by using DT due to more data | [44] |  | X |  | X |  |  |  |  |  |  |  |
|  | **Support for recovery and health (no relation to DT)** | A perceived safety is an important factor that contributes to patients’ recovery and health | [3] | X |  |  |  |  |  |  |  |  |  |  |
|  | **Enriching the community & better patient health due to DT** | DT initiatives are shown to benefit resources, enrich the community and improve care that ultimately serves to better patient health | [12] |  | X |  |  |  |  |  |  |  |  |  |
|  | **Faster access to care by using DT** | Facilitating symptom reporting in this manner consequently resulted in faster access to care | [7] |  |  | X |  |  |  |  |  |  |  |  |
|  | **Maintaining activities of daily living and increased independent living at home by using DT** | The use of DT is important so that older people stay living more independently at home and can maintain their activities | [43] |  | X |  |  |  |  |  |  |  |  |  |
|  | **Increased socialisation and conversation through DT** | DT could reduce the isolation risk of the elderly and improve the socialisation and conversation | [57] | X |  |  |  |  |  |  |  |  |  |  |
|  | **Freedom to express concerns about DT** | Patients could express their concerns more freely over DT | [40] |  | X |  |  |  |  |  |  |  |  |  |
|  | **Self-management and achievement of personal goals by using DT (contribute to well-being)** | Feeling safe when using DT can enable people to support their self-management and achieve their own health-related goals, which contributes to well-being | [57] |  |  | X |  |  |  |  |  |  |  |  |
| **Willingness to sacrifice privacy** | **Willingness to sacrifice privacy when using DT** | Sacrifice some privacy, for example by being supervised, to increase perceived safety | [6] |  |  |  |  |  | X |  |  |  |  |  |
| **Healthcare providers** | | | | | | | | | | | | | | |
| **Relief in using DTs** | | DT can decrease the caregivers’ burdens | [44] |  | X |  | X |  |  |  |  |  |  |  |
| **Both healthcare recipients and providers** | | | | | | | | | | | | | | |
| **Openness, improved problem discussion & improved mental effects by using DTs** | | Increased emotional safety leads to increased openness and problem discussion in online counselling | [55] |  |  |  |  |  |  |  |  |  | X |  |
|  |  | Emotional and physical safety—both with the therapist and within close relationships—are the foundations of being able to be open and vulnerable in healthy relationships | [47] |  | X |  |  |  |  |  |  |  |  |  |
| **Increased empowerment & participation in own therapy (also feelings of higher participation) by using DTs** | | One way to participate was to self-report symptoms using the DT | [7] |  |  | X |  |  |  |  |  |  |  |  |
| **Faster responsiveness of healthcare providers to changing parameters, problems, and questions from healthcare recipients by using DTs** | | Deeper information at hand for healthcare recipients by using DT | [48] |  |  |  | X |  |  |  |  |  |  |  |
|  |  | More proactive action in case of deterioration of vital parameters, as problems and questions could then be responded to immediately | [12] |  | X |  |  |  |  |  |  |  |  |  |
| **Relatives** | | | | | | | | | | | | | | |
| **Self-confidence when using DTs** | | Believe in their own capacity to take care of their child | [12] |  | X |  |  |  |  |  |  |  |  |  |
| **Increased involvement with informal caregivers when using DTs** | | Relatives who are involved in the care of older family members can become more involved in their daily lives using DT | [6] |  |  |  |  |  | X |  |  |  |  |  |
| **Opportunity of sharing care between informal caregivers by using DTs** | | Care can be shared between several relatives, as care is not dependent on just one person when using DT | [6] |  |  |  |  |  | X |  |  |  |  |  |
| **Improved support for relatives in stressful situations by using DTs** | | Less burden in the transition phase from hospital to home due to DT and the possibility to receive better support | [12] |  | X |  |  |  |  |  |  |  |  |  |
| **New care opportunity for informal caregivers or relatives by using DTs** (surveillance and respond remotely & communicate with caregivers when needed) | | Relatives could monitor dementia patients remotely and contact them by phone or go to their home in the case of an alarm. | [48] |  |  |  | X |  |  |  |  |  |  |  |
|  |  | Using DT results in opportunities to communicate earlier when needed | [12] |  | X |  |  |  |  |  |  |  |  |  |
| **Healthcare recipients and relatives** | | | | | | | | | | | | | | |
| **Enhanced peace of mind by using DTs** | | Using DT brings peace of mind for users and their families | [54] |  |  |  | X |  |  |  |  |  |  |  |
| **Implementation of DT** | | | | | | | | | | | | | | |
| **Willingness to adopt DTs in the workplace** | | Teams with high levels of psychological safety may be more likely to initiate telemedicine because they feel safe to take risks on behalf of the patient (e.g., using the device for the patient) | [51] |  | X |  |  |  |  |  |  |  |  |  |
| **Successful adoption of DTs*** | | An increased perceived safety is important for the successful adoption of telecare services among older adults | [43] |  | X |  |  |  |  |  |  |  |  |  |
| **Higher acceptance and adherence to DTs** | | Perceived safety is highly relevant for acceptance and adherence of the DT | [48] |  |  |  | X |  |  |  |  |  |  |  |
| **Increased use of DTs*** | | The [family] caregivers encourage using DT as they expect it to enhance safety | [44] |  | X |  | X |  |  |  |  |  |  |  |
| **Healthcare** | | | | | | | | | | | | | | |
| **Opportunity for earlier hospital discharge when using DTs** | | The opportunity to go home and still have the support through the DT was described as positive | [41] |  |  |  | X |  |  |  |  |  |  |  |
|  |  | The DT made it safer to leave the hospital knowing how easily it is to reach the nurses | [12] |  | X |  |  |  |  |  |  |  |  |  |
| **Minimising physical hospital visits by using DTs** | | Using DT could replace some of the physical visits and could minimise the need for hospitalisation | [12] |  | X |  |  |  |  |  |  |  |  |  |
| **Minimising emergency hospital visits by using DTs** | | Increased confidence in care, which may have contributed to reduced emergency hospital admissions, since patients felt supported at home | [7] |  |  | X |  |  |  |  |  |  |  |  |
|  |  | DT decreased the need of scheduled visits and the number of emergency visits to the hospital | [12] |  | X |  |  |  |  |  |  |  |  |  |
| **Improved healthcare services by using DTs** | | DT are shown to benefit resources, enrich the community and improve care that ultimately serves to better patient health | [12] |  | X |  |  |  |  |  |  |  |  |  |
| **Increased care recipient confidence in healthcare** | | Increased confidence in healthcare, which may have contributed to reduced emergency hospital admissions, since patients felt supported at home | [7] |  |  | X |  |  |  |  |  |  |  |  |
| **Economy** | | | | | | | | | | | | | | |
| **Sustainable development and efficient use of resources by using DTs*** | | DT are shown to benefit resources, enrich the community and improve care that ultimately serves to better patient health | [12] |  | X |  |  |  |  |  |  |  |  |  |
|  |  | At organizational and societal levels, DT may contribute to sustainable development and more efficient use of resources | [7] |  |  | X |  |  |  |  |  |  |  |  |
| **Cost savings for the healthcare system and care delivery by using DTs*** | | Older patients can age in place, and, at the same time, the health and welfare system can reduce care costs | [6] |  |  |  |  |  | X |  |  |  |  |  |
| **Negative outcomes of a strong perceived safety** | | | | | | | | | | | | | | |
| **Risk of too much data due to DTs** | | The fact that more data is produced and presented through DT can lead to too many data measurements due to the increased reliance on this data and the resulting increased need for safety | [52] |  |  |  | X |  |  |  |  |  |  |  |
| **Too much emotional attachment to DTs** | | If the quasi-human interaction leads to the projection of (too) intense emotions onto the robot, a technical malfunction, a dysfunctional failure of the technology could lead to depressive moods | [59] | X |  |  |  |  |  |  |  |  |  |  |
| **Risk of a false perceived safety related to DTs**  (perceived safety created by the transmission of data alone triggers a lack of mindfulness) | | There also seems to be a risk that when submitting data for interpretation by healthcare professionals, the act in itself may generate perceived safety, which unfortunately may result in ignoring warning signals and waiting for an intervention | [7] |  |  | X |  |  |  |  |  |  |  |  |
| **Promoting avoidance and defence strategies in engaging with the unknown through increased using DTs*** | | Because people feel too safe in online environments and DT provides such an online environment, people remain in avoidance and defence strategies and are not challenged to participate in active life and come into contact with the unfamiliar. | [55] |  |  |  |  |  |  |  |  |  | X |  |
| **Digital technology 1-11: 1 = Robotics; 2 = Telehealth; 3 = E-Health general; 4 = Telemonitoring; 5 = Digital apps on health management; 6 = Camera surveillance; 7 = Internet-based group platform; 8 =** **Digital personal health information management; 9 = Digital medicine dispenser; 10 = Online counselling; 11 = Participant simulation programme**  DT = Digital technology  * = Explicitly mentioned as an outcome | | | | | | | | | | | | | | |
