# Supplementary material for: Feeling safe in the context of digitalization in healthcare: a scoping review
Source: Syst Rev. 2024 Feb 8;13:62. doi: 10.1186/s13643-024-02465-9 (PMC10851492; doi:10.1186/s13643-024-02465-9)
Supplement: Supplementary file 3 — Additional file 3. Outcomes of a low perceived safety. [file 13643_2024_2465_MOESM3_ESM.docx]

**Additional file 3**

| **Additional file 3: Outcomes of a low perceived safety** | | | | | | | | | | | | | | |
| --- | --- | --- | --- | --- | --- | --- | --- | --- | --- | --- | --- | --- | --- | --- |
| **Outcomes of a low perceived safety** | | **Context of Outcomes** | **Source** | **Digital technologies** | | | | | | | | | | |
|  | |  |  | **1** | **2** | **3** | **4** | **5** | **6** | **7** | **8** | **9** | **10** | **11** |
| **Healthcare recipients** | | | | | | | | | | | | | | |
| **Fears and doubts concerning DTs** | | Fears and doubts concerning such technology, as well as integrity issues | [6] |  |  |  |  |  | X |  |  |  |  |  |
| **Sense of failure when using DTs** | | The need to ask for help and a sense of failure may make older adults feel more vulnerable and even humiliated | [9] |  |  | X |  |  |  |  |  |  |  |  |
| **Competencies and capacities in health management of healthcare recipients** | **Reduced capacity for effective disease management when using DT** | Low emotional safety in using DT can affect a patient's ability to engage in effective disease management | [37] |  |  |  |  |  |  |  | X |  |  |  |
|  | **Difficulty in filtering important information generated by DTs** | The vast amount of information generated by DT might be difficult to filter, especially for people with limited digital literacy | [9] |  |  | X |  |  |  |  |  |  |  |  |
|  | **Increased dependency due to increased need for assistance in using DTs** | Introducing technology [in situations when patients are exceptionally dependent, have cognitive difficulties or are old and frail] instead of empowering patients could make the patient even more dependent for instance on informal care | [2] |  |  |  |  |  |  |  |  | X |  |  |
| **Healthcare providers** | | | | | | | | | | | | | | |
| **Fear of loss of social contact if using DTs** | | Reduced social contact because of using DT worries healthcare providers | [9] |  |  | X |  |  |  |  |  |  |  |  |
| **Fear of incomplete digital information transfer and lack of holistic view of patient by DTs** | | The use of DT has the potential to misrepresent or incompletely portray the human aspect of medical communication | [9] |  |  | X |  |  |  |  |  |  |  |  |
| **Relatives** | | | | | | | | | | | | | | |
| **Uncertainty and ambivalence about letting older adults live at home alone** | | The uncertainty and the burden relatives experienced led to an ambivalent feeling between fulfilling the older adults’ last wish to live at home and the relief that a move to a nursing home would bring | [44] |  | X |  | X |  |  |  |  |  |  |  |
| **Role confusion for informal carers if they do not feel safe in dealing with DTs** | | The family members also reflected upon their role when receiving the alarms and messages, highlighting the risks of role confusion | [48] |  |  |  | X |  |  |  |  |  |  |  |
| **Implementation of DT** | | | | | | | | | | | | | | |
| **Non-use of DTs*** | | Older adults will stop using DT if the systems do not meet their needs | [44] |  | X |  | X |  |  |  |  |  |  |  |
| **Use of traditional face-to-face care and non-use of DTs*** | | Where concerns outweigh the priorities of the healthcare recipients benefits (e.g., building a doctor/patient relationship), patients will likely continue to seek in-person care | [60] |  | X |  |  |  |  |  |  |  |  |  |
| **Healthcare** | | | | | | | | | | | | | | |
| **Loss of trust in healthcare and government due to a dysfunctional DTs*** | | On a larger scale, this may affect trust in the health system and in governments | [57] |  |  | X |  |  |  |  |  |  |  |  |
| **Rejection of DTs in healthcare*** | | People perceived that robots could be of use in industry, but not in healthcare | [3] | X |  |  |  |  |  |  |  |  |  |  |
| **Fear of dehumanising healthcare** | | Participants were fearful that the introduction of humanoid robots into the care setting would lead to a loss of human contact and thus to dehumanised healthcare | [3] | X |  |  |  |  |  |  |  |  |  |  |
|  |  | DTs can lead to a loss of personal contact in the application in contrast to conventional healthcare service provision | [9] |  |  | X |  |  |  |  |  |  |  |  |
|  |  | Perceived unsafety concerns the lost opportunity for direct dialogue with healthcare providers due to primary use of DT | [9] |  |  | X |  |  |  |  |  |  |  |  |
| **Digital technology 1-11: 1 = Robotics; 2 = Telehealth; 3 = E-Health general; 4 = Telemonitoring; 5 = Digital apps on health management; 6 = Camera surveillance; 7 = Internet-based group platform; 8 =** **Digital personal health information management; 9 = Digital medicine dispenser; 10 = Online counselling; 11 = Participant simulation programme**  DT = Digital technology  * = Explicitly mentioned as an outcome | | | | | | | | | | | | | | |
