# Supplementary material for: Feeling safe in the context of digitalization in healthcare: a scoping review
Source: Syst Rev. 2024 Feb 8;13:62. doi: 10.1186/s13643-024-02465-9 (PMC10851492; doi:10.1186/s13643-024-02465-9)
Supplement: Supplementary file 4 — Additional file 4. Influencing factors and needs in the context of perceived safety and digital technologies in healthcare. [file 13643_2024_2465_MOESM4_ESM.docx]

**Additional file 4**

| **Additional File 4: Influencing factors and needs in the context of perceived safety and digital technologies in healthcare** | | | | | | | | | | | | | | | |
| --- | --- | --- | --- | --- | --- | --- | --- | --- | --- | --- | --- | --- | --- | --- | --- |
| **Core dimension** | **Main categories** | **IF** | **Source** | **Digital technologies** | | | | | | | | | | | **TG** |
|  |  |  |  | **1** | **2** | **3** | **4** | **5** | **6** | **7** | **8** | **9** | **10** | **11** |  |
| **Noticeable changes in care** due to DTs | Better healthcare provider responsiveness due to DT | + | [41] |  |  |  | X |  |  |  |  |  |  |  | HCR |
|  | DT enables efficient access to care (participation, empowerment) | + | [7];[49] |  |  | X |  | X |  |  |  |  |  |  | HCR |
|  | DT improves or speeds up care | + | [6];[7];[12];[40];[41];[42]; [44];[47];[55] |  | X | X | X |  | X |  |  |  | X |  | HCR, HCP |
|  | Innovative solutions to overcome communication barriers due to DT use | + | [3];[12];[41];[56] | X | X |  | X |  |  |  |  |  |  |  | HCR, HCP |
|  | Timely, efficient response possible through DT (e.g., deteriorating health) | + | [12] |  | X |  |  |  |  |  |  |  |  |  | HCP |
|  | DT to support (timely) medical treatment (in the event of a disaster) | + | [42];[51] |  | X |  |  |  |  |  |  |  |  |  | HCP |
|  | Lack of access to DT | - | [45] |  |  | X |  |  |  |  |  |  |  |  | HCR, FM |
|  | Difficulties in understanding unfamiliar digital communication | - | [3];[9];[39];[47];[50] | X | X | X |  |  |  |  |  |  |  |  | HCR |
|  | Perception of limited digital communication between healthcare providers and recipients | - | [9];[38];[40];[60] |  | X | X |  |  |  |  |  |  |  |  | HCR, HCP |
|  | Waiting too long for feedback on one’s health data (from healthcare providers or digitally) | - | [41];[48] |  |  |  | X |  |  |  |  |  |  |  | HCR |
|  | Anxiety about loss of human interaction | - | [3];[6];[9];[39];[41];[43];[55]; [57];[58];[60] | X | X | X | X |  | X |  |  |  | X |  | HCR, HCP |
|  | Need for human relationships^1^ | NB | [9];[36];[44] |  | X | X | X |  |  | X |  |  |  |  | HCR, HCP |
|  | Anticipated diagnostic and treatment errors due to DT (fear) | - | [36];[38] |  | X |  |  |  |  | X |  |  |  |  | HCP |
|  | Lack of interpersonal relationships and interaction through DT (concern) | - | [38] |  | X |  |  |  |  |  |  |  |  |  | HCP |
|  | Limited access to DT for vulnerable patient groups (concern) | - | [38];[42] |  | X |  |  |  |  |  |  |  |  |  | HCP |
|  | Providing easy access to DT^3^ | NB | [36];[42] |  | X |  |  |  |  | X |  |  |  |  | HCR, HCP |
|  | Healthcare recipient evaluates his or her individual needs and weighs the advantages and disadvantages of using DT^1^ | NE | [44] |  | X |  | X |  |  |  |  |  |  |  | HCR |
| **Digital (health) literacy** | Strong digital health literacy | + | [9];[41];[43] |  | X | X | X |  |  |  |  |  |  |  | HCR |
|  | Technology affinity of healthcare recipients | + | [39] | X |  |  |  |  |  |  |  |  |  |  | HCR |
|  | Healthcare recipients' knowledge about robots (not film knowledge) | + | [3] | X |  |  |  |  |  |  |  |  |  |  | HCR |
|  | Learning support for the acquisition of competencies by the healthcare recipients or their relatives | + | [39];[43];[57] | X | X | X |  |  |  |  |  |  |  |  | HCR, FM |
|  | Providing need-oriented information | + | [43] |  | X |  |  |  |  |  |  |  |  |  | HCR |
|  | Increased need for information before using digital technology^1^ | NB | [6];[9];[42];[43];[44] |  | X | X | X |  | X |  |  |  |  |  | HCR, HCP |
|  | Awareness of the limitations of DT | + | [43] |  | X |  |  |  |  |  |  |  |  |  | HCR |
|  | Awareness of DT skills requirements (basic knowledge/specifics of digital communication) | + | [43] |  | X |  |  |  |  |  |  |  |  |  | HCP |
|  | Education and training of healthcare providers in DT (as needed) | + | [60] |  | X |  |  |  |  |  |  |  |  |  | HCP |
|  | Knowledge of one's own performance limits when using DT^1^ | NE | [49] |  |  |  |  | X |  |  |  |  |  |  | HCR |
|  | Lack of digital literacy | - | [3];[6];[7];[9];[38];[43];[46]; [48];[49];[57] | X | X | X | X | X | X |  |  |  |  |  | HCR, HCP |
|  | Lack of or inadequate knowledge transfer/operating instructions | - | [12];[43];[44];[57] |  | X |  | X |  |  |  |  |  |  |  | HCR, FM |
|  | Knowledge regarding high technological demands on users of DT (amount of data, speed of (further) development) | - | [9];[38];[43];[52];[57] |  | X | X | X |  |  |  |  |  |  |  | HCR, HCP |
|  | Knowledge regarding changing complex mental and physical demands on healthcare providers due to DT | - | [36];[58] |  |  | X |  |  |  | X |  |  |  |  | HCP |
|  | Lack of or limited skills/knowledge/competencies of DT | - | [2];[39];[42] | X | X |  |  |  |  |  |  | X |  |  | HCP |
|  | Limited transferability of previous competencies and skills into a digital setting | - | [38];[42] |  | X |  |  |  |  |  |  |  |  |  | HCP |
| **Design & appearance** of DTs | Simple design/handling of the DT | + | [55] |  |  |  |  |  |  |  |  |  | X |  | HCR |
|  | DT has a feedback system | + | [41];[46] |  |  |  | X |  |  |  |  |  |  |  | HCR |
|  | Human design of DT | + | [3] | X |  |  |  |  |  |  |  |  |  |  | HCR |
|  | Non-human design of DT | + | [3] | X |  |  |  |  |  |  |  |  |  |  | HCR |
|  | Participation of healthcare recipients in the development of DT (Co-Design) | + | [3];[12];[37];[39];[43];[46]; [52];[60] | X | X |  | X |  |  |  | X |  |  |  | HCR |
|  | Visibility and invisibility of DT | + | [3];[6];[44];[46];[54] | X | X |  | X |  | X |  |  |  |  |  | HCR |
|  | No additional devices need to be used^2^ | NE | [12] |  | X |  |  |  |  |  |  |  |  |  | HCR |
|  | No adequate (too much or too little) humanisation of DT | - | [3];[59] | X |  |  |  |  |  |  |  |  |  |  | HCR |
|  | No physical examinations possible due to DT | - | [40] |  | X |  |  |  |  |  |  |  |  |  | HCR |
|  | DT as a (physical) disruptor | - | [3];[6];[7];[38];[40]; [47] | X | X | X |  |  |  |  |  |  |  |  | HCR |
| **Need-oriented technology** | Designing DT based on needs, expectations and values (also within the design process) (reassurance) | + | [2];[3];[6];[9];[37];[40];[41]; [43];[45];[46];[49];[54];[55]; [58];[60] | X | X | X | X | X | X |  | X | X | X |  | HCR, HCP, FM |
|  | Importance of user participation as co-designers during development phase^3^ | NB | [36];[41];[52];[58] |  |  | X | X |  |  | X |  |  |  |  | HCR, HCP |
|  | Conduct a user needs analysis prior to the implementation of DT^3^ | NB | [43];[44];[52] |  | X |  | X |  |  |  |  |  |  |  | HCR, HCP |
|  | Customising the functions and features^2^ | NB | [3];[36];[37];[41];[44];[48]; [52] | X | X |  | X |  |  | X | X |  |  |  | HCR, HCP |
|  | Different individual functions of the DT^2^ | NE | [48];[52];[54] |  |  |  | X |  |  |  |  |  |  |  | HCR, FM |
|  | Deal with emergency situations^2^ | NB | [36] |  |  |  |  |  |  | X |  |  |  |  | HCR, HCP |
|  | Targeted DT offerings based on health conditions | + | [44] |  | X |  | X |  |  |  |  |  |  |  | HCR, FM |
|  |  | + | [2];[43];[54] |  | X |  | X |  |  |  |  | X |  |  | HCP, HCR |
|  | Choice of DT according to needs of healthcare recipients (increase of autonomy) | + | [2];[43] |  | X |  |  |  |  |  |  | X |  |  | HCP |
|  | Individual patient views regarding the design of the DT should be considered | +/- | [3] | X |  |  |  |  |  |  |  |  |  |  | HCR |
|  | Considering individual patient views regarding the potentials of the DT | +/- | [3] | X |  |  |  |  |  |  |  |  |  |  | HCR |
|  | Considering individual patient views regarding human characteristics of DT | +/- | [3] | X |  |  |  |  |  |  |  |  |  |  | HCR |
|  | Lack of orientation of DT towards the needs of the recipients | - | [46] |  |  |  | X |  |  |  |  |  |  |  | HCR, HCP |
| **Support** | Presence of healthcare provider when using DT | + | [3];[43] | X | X |  |  |  |  |  |  |  |  |  | HCR |
|  | Healthcare recipients feel that healthcare providers trust the DT | + | [53] |  |  | X |  |  |  |  |  |  |  |  | HCR |
|  | Digital opportunity for communication between healthcare recipients and healthcare providers about digital data | + | [12];[41];[49] |  | X |  | X | X |  |  |  |  |  |  | HCR |
|  | Approachability of healthcare professionals and service staff to DT | + | [6];[52];[56] |  | X |  | X |  | X |  |  |  |  |  | HCR |
|  | Opportunity of (efficient) support through DT if required | + | [6];[9];[12];[44];[46];[48];[54] |  | X | X | X |  | X |  |  |  |  |  | HCR |
|  | Getting support if needed^1^ | NE | [52] |  |  |  | X |  |  |  |  |  |  |  | HCR |
|  | Healthcare recipients require a high level of expertise from healthcare providers to get support^1^ | NE | [44] |  | X |  | X |  |  |  |  |  |  |  | HCR |
|  | Coaching and reflection on the use of DT | + | [60] |  | X |  |  |  |  |  |  |  |  |  | HCP |
|  | Feedback from healthcare recipients to healthcare providers in order to improve the quality-of-service delivery^1^ | NP | [42] |  | X |  |  |  |  |  |  |  |  |  | HCP |
|  | Availability of trained staff | + | [35] |  |  |  |  |  |  |  |  |  |  | X | HCP |
|  | Lacking support when technical problems occur | - | [48] |  |  |  | X |  |  |  |  |  |  |  | HCR |
| **Trust** | Efficiency, predictability & reliability in DT (builds trust) | + | [3];[40] | X | X |  |  |  |  |  |  |  |  |  | HCR |
|  | DT that promotes equal, individualised, and non-biased care | + | [3];[7];[47] | X | X | X |  |  |  |  |  |  |  |  | HCR |
|  | Equal distribution of power between healthcare providers and recipients in DT, e.g., in online consultations | + | [55];[57] |  |  | X |  |  |  |  |  |  | X |  | HCR |
|  | Acceptance of DT as an integral part of the clinical team | + | [46] |  |  |  | X |  |  |  |  |  |  |  | HCR |
|  | Positive attitude towards DT in the workplace | + | [53] |  |  | X |  |  |  |  |  |  |  |  | HCP |
|  | Developing a confidential relationship between healthcare recipient/provider | + | [41];[56];[60] |  | X |  | X |  |  |  |  |  |  |  | HCP, HCR |
|  | More professional distance between healthcare provider and recipients through DT (in psychological settings) | + | [55] |  |  |  |  |  |  |  |  |  | X |  | HCP |
|  | Lack of confidence in DT to perform complex tasks | - | [3] | X |  |  |  |  |  |  |  |  |  |  | HCR |
|  | Negative general attitude towards DT (e.g., "cold and distant" technology) | - | [2] |  |  |  |  |  |  |  |  | X |  |  | HCR |
|  | Expectation that healthcare providers will misuse the information entered in the DT | - | [49] |  |  |  |  | X |  |  |  |  |  |  | HCR |
|  | Limited openness of healthcare recipients to DT (more difficult diagnosis due to DT) | - | [38] |  | X |  |  |  |  |  |  |  |  |  | HCP, HCR |
| **Degree of privacy** in relation to the DTs | Security-related privacy and data protection trade-offs | +/- | [48] |  |  |  | X |  |  |  |  |  |  |  | FM |
|  | Risk/benefit trade-off (privacy/benefit) | +/- | [54] |  |  |  | X |  |  |  |  |  |  |  | HCR |
|  | Lack of privacy | - | [6];[7];[9];[40];[44];[47];[52]; [56];[60] |  | X | X | X |  | X |  |  |  |  |  | HCR, HCP |
|  | Need for privacy^1^ | NB | [36];[47];[59] | X | X |  |  |  |  | X |  |  |  |  | HCR, HCP |
|  | DT shared by several patients (inhibition to express problems) | - | [36] |  |  |  |  |  |  | X |  |  |  |  | HCR |
|  | Need for recipients to be anonymous^1^ | NE | [36];[55] |  |  |  |  |  |  | X |  |  | X |  | HCR |
| **Noticeable changes in social life** due to DTs | Enabling participation in social life through DT | + | [6];[12];[43];[44];[57] |  | X | X | X |  | X |  |  |  |  |  | HCR, FM |
|  | Use of DT in the home (providing a safe space, anonymity) | + | [40];[47];[55];[57]; [60] |  | X | X |  |  |  |  |  |  | X |  | HCR |
|  | DT allows people to stay at home | + | [40];[47];[60] |  | X |  |  |  |  |  |  |  |  |  | HCR |
|  | DT enables the possibility of independence or remaining at home^3^ | NE | [44] |  | X |  |  |  |  |  |  |  |  |  | HCR |
|  | DT promotes human contact between healthcare recipients/providers | + | [3] | X |  |  |  |  |  |  |  |  |  |  | HCR |
|  | Unconscious role of caring relatives in using DT (too much responsibility) | - | [6];[48] |  |  |  | X |  | X |  |  |  |  |  | FM |
| **Technical weaknesses/deficiencies** | Technical weaknesses/deficiencies | - | [3];[6];[9];[41];[42];[43];[44]; [45];[48] | X | X | X | X |  | X |  |  |  |  |  | HCR, HCP |
|  | Functional readiness by testing extensively before DT is used in practice^2^ | NB | [36];[43] |  | X |  |  |  |  | X |  |  |  |  | HCR, HCP |
| **Control of and due to DTs** ‘sense of control’ | Supervision of DT by medical staff (no independent decisions by DT) | + | [3] | X |  |  |  |  |  |  |  |  |  |  | HCR |
|  | Control options via DT for termination | + | [55] |  |  |  |  |  |  |  |  |  | X |  | HCR |
|  | Controlling and accessing own data (data sovereignty) | + | [3];[7];[45] | X |  | X |  |  |  |  |  |  |  |  | HCR |
|  | Health monitoring through DT (feeling of protection) | + | [6];[46];[54] |  |  |  | X |  | X |  |  |  |  |  | HCR |
|  | DT replaces human control | + | [44] |  | X |  | X |  |  |  |  |  |  |  | HCR |
|  | DT are used to give clear signals that they are currently functioning and working^2^ | NE | [44] |  | X |  | X |  |  |  |  |  |  |  | HCR |
|  | Comprehensive data access rights for healthcare providers to use DT | - | [45] |  |  | X |  |  |  |  |  |  |  |  | HCP |
|  | Autonomous decision-making options when using DT^1^ | NE | [36];[55] |  |  |  |  |  |  | X |  |  | X |  | HCR |
| **Data security** | Lacking data security | - | [3];[9];[41];[45]; [49];[57] | X |  | X | X | X |  |  |  |  |  |  | HCR, FM, HCP |
|  | Concerns about data protection and data security for healthcare recipients | - | [38] |  | X |  |  |  |  |  |  |  |  |  | HCP, HCR |
| **Transparency** | Transparency of DT (personal health data & data processing) | + | [3];[9] | X |  | X |  |  |  |  |  |  |  |  | HCR |
|  | Transparency of your data | + | [7];[45];[46];[48] |  |  | X | X |  |  |  |  |  |  |  | HCR, FM |
|  | Lack of transparency/clarity (technical data transfer processes) | - | [9];[41] |  |  | X | X |  |  |  |  |  |  |  | HCR |
| **Health and psychosocial conditions** | Health and psychosocial conditions for being able to use DT | - | [3];[43];[50] | X | X | X |  |  |  |  |  |  |  |  | HCR, HCP |
|  | Limitations in the use of DT due to a medical condition or disease | - | [3];[38];[42];[43];[48] | X | X |  | X |  |  |  |  |  |  |  | HCR, HCP, FM |
|  | Early implementation of DT (in case of progressive deterioration of illness)^3^ | NE | [48] |  |  |  | X |  |  |  |  |  |  |  | FM |
| **Demands on the users** | Negative feelings (stress, anxiety) when using DT | - | [9];[52] |  |  | X | X |  |  |  |  |  |  |  | HCR |
|  | Health apps set targets too high | - | [49] |  |  |  |  | X |  |  |  |  |  |  | HCR |
|  | Sudden change in the procedure for claiming medical services ("Rapid changes" in society and high expectations of society) | - | [9] |  |  | X |  |  |  |  |  |  |  |  | HCR |
|  | High social pressure on healthcare recipients regarding use and digital health literacy in relation to DT | - | [9] |  |  | X |  |  |  |  |  |  |  |  | HCR |
|  | Uncomfortable with the demands for assistance in using DT | - | [9] |  |  | X |  |  |  |  |  |  |  |  | HCR |
| **Status of scientific knowledge** | Evidence-based needs of specific (vulnerable) patient groups related to DT | + | [52];[60] |  | X |  | X |  |  |  |  |  |  |  | HCR, HCP |
|  | Research gaps on whether anthropomorphic handling of robots in healthcare relationships is advisable or not | +/- | [59] | X |  |  |  |  |  |  |  |  |  |  | HCR |
| **Ethical challenges** | Ethical issues | - | [48] |  |  |  | X |  |  |  |  |  |  |  | HCR, HCP, FM |
|  | Unresolved ethical issues | - | [9];[48] |  |  | X | X |  |  |  |  |  |  |  | HCR, HCP |
|  | Fulfilment of basic ethical needs^1^ | NB | [58] |  |  | X |  |  |  |  |  |  |  |  | HCR, HCP |
|  | Human-centred approach^2^ | NB | [9];[58] |  |  | X |  |  |  |  |  |  |  |  | HCR, HCP |
| **Financial uncertainties** | Uncertainty among healthcare providers about funding for DT | - | [36];[42] |  | X |  |  |  |  | X |  |  |  |  | HCP |
| **Experiences and routines** | Successful, routine and standardised entry into DT | + | [38] |  | X |  |  |  |  |  |  |  |  |  | HCP |
|  | Experience using DT | + | [43] |  | X |  |  |  |  |  |  |  |  |  | HCP |
|  | Practical, individualised exercises in the use of digital technology^1^ | NB | [43] |  | X |  |  |  |  |  |  |  |  |  | HCR, HCP |
|  | Sufficient time before the first use^3^ | NE | [12] |  | X |  |  |  |  |  |  |  |  |  | HCR |
| **Organizational culture** | Positive organizational culture when implementing a new DT (psychological well-being) | + | [53] |  |  | X |  |  |  |  |  |  |  |  | HCP |
| ***Initial face-to-face contact*** | *Initial face-to-face contact before the first use of digital technology in order to better assess the healthcare recipient^1^* | *NP* | *[42]* |  | *X* |  |  |  |  |  |  |  |  |  | HCP |
| ***Creating the contextual conditions for DTs*** | *Creating the contextual conditions for digital technology (e.g., creating a calm environment, organizational & political support for implementation)^3^* | *NB* | *[38];[42];[47]* |  | *X* |  |  |  |  |  |  |  |  |  | *HCR, HCP* |
|  | *Reducing barriers to usage/ensuring integration into existing workflows^3^* | *NB* | *[51]* |  | *X* |  |  |  |  |  |  |  |  |  | *HCR, HCP* |
| **Digital technology 1-11:** 1 = Robotics; 2 = Telehealth; 3 = E-Health general; 4 = Telemonitoring; 5 = Digital apps on health management; 6 = Camera surveillance; 7 = Internet-based group platform; 8 = Digital personal health information management; 9 = Digital medicine dispenser; 10 = Online counselling; 11 = Participant simulation programme  TG = Target group; DT = Digital technology; NE = Needs in the context of emotional safety; NP = Needs in the context of psychological safety; NB = Needs in the context of emotional and psychological safety (^1^ = Needs regarding the stakeholders themselves; ^2^ = Needs regarding the DT; ^3^ = Needs regarding the environment)  **Perspectives of the target groups (TG):** HCP = healthcare provider; HCR = healthcare recipient; FM = family members  **IF = Influencing factors; + = Facilitating factors; - = Inhibitory factors; +/- = Both facilitating and inhibitory factors** | | | | | | | | | | | | | | | |
