# Supplementary material for: Feeling safe in the context of digitalization in healthcare: a scoping review
Source: Syst Rev. 2024 Feb 8;13:62. doi: 10.1186/s13643-024-02465-9 (PMC10851492; doi:10.1186/s13643-024-02465-9)
Supplement: Supplementary file 5 — Additional file 5. Perspectives on emotional or psychological safety (explicit mentioned in included literature). [file 13643_2024_2465_MOESM5_ESM.docx]

**Additional File 5**

| **Additional File 5: Perspectives on emotional or psychological safety (explicit mentioned in included literature)** | | | | | | | | | | | | | |
| --- | --- | --- | --- | --- | --- | --- | --- | --- | --- | --- | --- | --- | --- |
| **Main category** | **Context of perspectives** | **Source** | **Digital technologies** | | | | | | | | | | |
|  |  |  | **1** | **2** | **3** | **4** | **5** | **6** | **7** | **8** | **9** | **10** | **11** |
| **Ethical** | | | | | | | | | | | | | |
| **Ethical framework has not changed due to DTs** | DTs have not changed our ethical framework in terms of the fundamental values, rights and freedoms enshrined in the Constitution and the European Charter of Fundamental Rights | [58] |  |  | X |  |  |  |  |  |  |  |  |
| **Ethical capacity to act** | All people involved in using the service must know how to use and handle the technology in a practical and ethical way | [6] |  |  |  |  |  | X |  |  |  |  |  |
| **Ensure that healthcare providers' physical and psychological integrity is not at risk** | The physical and emotional safety of humans and possible ethical consequences in the operation and use of algorithmic systems must also be considered | [58] |  |  | X |  |  |  |  |  |  |  |  |
| **Careful reflection on the benefits of technology compared to potential disadvantages and ethical implications** | Careful reflection on the benefits of technology compared to potential disadvantages and ethical implications is crucial | [2] |  |  |  |  |  |  |  |  | X |  |  |
| **Ethical concerns about the over-humanisation of DTs** | If the quasi-human interaction leads to the projection of (too) intense emotions onto the robot, a technical malfunction, a dysfunctional failure of the DT could lead to depressive moods | [59] | X |  |  |  |  |  |  |  |  |  |  |
| **Research gap on whether and to what extent a humanising approach to DTs in care relationships is advisable or contraindicated** | A general rule for the question of whether and to what extent an anthropomorphising approach to robots in care relationships is advisable or contraindicated cannot be derived from the findings to date | [59] | X |  |  |  |  |  |  |  |  |  |  |
| **Ethical issues related to privacy** | Ethical issues were raised in terms of the privacy of the person living with dementia | [48] |  |  |  | X |  |  |  |  |  |  |  |
| **Safety issues more important than privacy for the majority** | Ethical issues were raised in terms of the privacy of the person living with dementia but for the majority, safety issues were more important than privacy | [48] |  |  |  | X |  |  |  |  |  |  |  |
| **Being thrown into a situation unprepared is unethical** | Loss of dignity by being thrown into a situation in this way is unethical and especially noticed in its rupture | [9] |  |  | X |  |  |  |  |  |  |  |  |
| **Economic** | | | | | | | | | | | | | |
| **Economic and practical benefits through perceived safety and the dT** | eHomecare is expected to provide economic and practical benefits to society, while at the same time the older adult will have the opportunity to age in place safely | [6] |  |  |  |  |  | X |  |  |  |  |  |
| **Economic and time savings are cited as the main benefits of using DTs** | Economic and time-saving benefits are often mentioned by clients as advantages for their participation in teletherapy | [47] |  | X |  |  |  |  |  |  |  |  |  |
|  | Maintaining funding for GPNs to provide telehealth services was considered important to facilitate better use of their roles and to ensure that health assessments and other preventive or health promotion initiatives were maintained | [42] |  | X |  |  |  |  |  |  |  |  |  |
| **Non-acceptance of DTs due to cost** | Cost has been mentioned as a common reason for not accepting the DT | [6] |  |  |  |  |  | X |  |  |  |  |  |
| **Main barriers to acquiring digital literacy skills are financial costs** | As the main obstacles to teletherapy training, the financial costs are discussed | [47] |  | X |  |  |  |  |  |  |  |  |  |
| **Reducing purchase costs and barriers to the use of telehealth services on recipients' personal devices** | If participants use their own digital devices, this results in lower acquisition costs for the hospital and easier access for participants | [12] |  | X |  |  |  |  |  |  |  |  |  |
| **A lack of financial concerns allows the new technology to be trialled and overrides other concerns about acceptance of DTs** | If there are no financial concerns, the willingness to try a new DT can crowd out other concerns that affect the acceptance of the DT | [6] |  |  |  |  |  | X |  |  |  |  |  |
| **Political** | | | | | | | | | | | | | |
| **Socio-technical complexities need to be addressed politically to ensure the success of DT implementation** | A political agenda must support this by recognising the socio-technical complexity that determines the success of DT implementation | [46] |  |  |  | X |  |  |  |  |  |  |  |
| **Policy makers are challenged to think about how DTs are managed at individual and societal level** | Need for a better understanding of the importance of self-management and well-being. Researchers, policy makers and people are challenged to think about how we - on an individual and societal level - engage with DTs | [57] |  |  | X |  |  |  |  |  |  |  |  |
| **Policy makers need to tailor the process of DT implementation to the different user groups** | The process of DT adoption must be tailored to the different user groups, with a focus on policy makers as well | [48] |  |  |  | X |  |  |  |  |  |  |  |
| **Psychosocial/society** | | | | | | | | | | | | | |
| **Increased social network** | A benefit for the older patient could be an increased social network | [6] |  |  |  |  |  | X |  |  |  |  |  |
| **Healthcare recipients may face increased risk to their safety simply because of the desire to remain at home “at any cost”** | Patients may face increased risk to their safety simply because of the desire to remain at home “at any cost” | [37] |  |  |  |  |  |  |  | X |  |  |  |
| **Enable communication with other people and train the ability to express needs and live accordingly** | It may favour the opening of the person in need of care for communication also with other people, train and vitalise the spectrum of their emotional possibilities, strengthen their perceived safety in the awareness of a reliable "coaching" of their needs and produce other mental effects that are also beneficial | [59] | X |  |  |  |  |  |  |  |  |  |  |
| **Legal** | | | | | | | | | | | | | |
|  | No quotes found |  |  |  |  |  |  |  |  |  |  |  |  |
| **Digital technology 1-11:** 1 = Robotics; 2 = Telehealth; 3 = E-Health general; 4 = Telemonitoring; 5 = Digital apps on health management; 6 = Camera surveillance; 7 = Internet-based group platform; 8 = Digital personal health information management; 9 = Digital medicine dispenser; 10 = Online counselling; 11 = Participant simulation programme  DT = Digital technology | | | | | | | | | | | | | |
